# Sweat bees on hot chillies: provision of pollination services by native bees in traditional slash‐and‐burn agriculture in the Yucatán Peninsula of tropical Mexico

**DOI:** 10.1111/1365-2664.12860

**Published:** 2017-01-27

**Authors:** Patricia Landaverde‐González, José Javier G. Quezada‐Euán, Panagiotis Theodorou, Tomás E. Murray, Martin Husemann, Ricardo Ayala, Humberto Moo‐Valle, Rémy Vandame, Robert J. Paxton

**Affiliations:** ^1^ General Zoology Institute for Biology Martin Luther University Halle‐Wittenberg Halle (Saale) Germany; ^2^ Department of Computational Landscape Ecology UFZ – Helmholtz Centre for Environmental Research Leipzig Germany; ^3^ Departamento de Apicultura Tropical Campus Ciencias Biológicas y Agropecuarias Universidad Autónoma de Yucatán Mérida México; ^4^ German Centre for Integrative Biodiversity Research (iDiv) Halle‐Jena‐Leipzig Leipzig Germany; ^5^ National Biodiversity Data Centre Waterford Ireland; ^6^ Estación de Biología Chamela (Sede Colima) Instituto de Biología Universidad nacional Autónoma de México San Patricio Jalisco México; ^7^ Departamento Agricultura Sociedad y Ambiente El Colegio de la Frontera Sur Carretera Panamericana y Periférico Sur s/n María Auxiliadora Chiapas México

**Keywords:** bee abundance, bee richness, biodiversity, ecosystem service, Habanero chilli, land use, *Lasioglossum*, *milpa*, slash‐and‐burn, sweat bee

## Abstract

1. Traditional tropical agriculture often entails a form of slash‐and‐burn land management that may adversely affect ecosystem services such as pollination, which are required for successful crop yields. The Yucatán Peninsula of Mexico has a >4000 year history of traditional slash‐and‐burn agriculture, termed ‘milpa’. Hot ‘Habanero’ chilli is a major pollinator‐dependent crop that nowadays is often grown in monoculture within the *milpa* system.
2. We studied 37 local farmers’ chilli fields (sites) to evaluate the effects of landscape composition on bee communities. At 11 of these sites, we undertook experimental pollination treatments to quantify the pollination of chilli. We further explored the relationships between landscape composition, bee communities and pollination service provision to chilli.
3. Bee species richness, particularly species of the family Apidae, was positively related to the amount of forest cover. Species diversity decreased with increasing proportion of crop land surrounding each sampling site. Sweat bees of the genus *Lasioglossum* were the most abundant bee taxon in chilli fields and, in contrast to other bee species, increased in abundance with the proportion of fallow land, gardens and pastures which are an integral part of the *milpa* system.
4. There was an average pollination shortfall of 21% for chilli across all sites; yet the shortfall was unrelated to the proportion of land covered by crops. Rather, chilli pollination was positively related to the abundance of *Lasioglossum* bees, probably an important pollinator of chilli, as well indirectly to the proportion of fallow land, gardens and pastures that promote *Lasioglossum* abundance.
5. *Synthesis and applications*. Current, low‐intensity traditional slash‐and‐burn (*milpa*) agriculture provides *Lasioglossum* spp. pollinators for successful chilli production; fallow land, gardens and pasture therefore need to be valued as important habitats for these and related ground‐nesting bee species. However, the negative impact of agriculture on total bee species diversity highlights how agricultural intensification is likely to reduce pollination services to crops, including chilli. Indeed, natural forest cover is vital in tropical Yucatán to maintain a rich assemblage of bee species and the provision of pollination services for diverse crops and wild flowers.

## Abstract 2

Current, low‐intensity traditional slash‐and‐burn (*milpa*) agriculture provides *Lasioglossum* spp. pollinators for successful chilli production; fallow land, gardens and pasture therefore need to be valued as important habitats for these and related ground‐nesting bee species. However, the negative impact of agriculture on total bee species diversity highlights how agricultural intensification is likely to reduce pollination services to crops, including chilli. Indeed, natural forest cover is vital in tropical Yucatán to maintain a rich assemblage of bee species and the provision of pollination services for diverse crops and wild flowers.

## Introduction

Pollination directly or indirectly contributes to the production of 75% of world crops, representing an economic value of €153 billion annually and contributing to 9·5% of world agricultural economic output (Klein *et al*. 2007; Gallai *et al*. 2009). Globally, 87·5% of all plants, agricultural and wild, are pollinated by animals (Ollerton, Winfree & Tarrant 2011). This makes animal‐mediated pollination one of the most important biotic interactions in terrestrial ecosystems, not only for ecosystem function but also for the maintenance of biodiversity. Bees are the most important animal pollinator group (Klein *et al*. 2007). Among the about 20 000 bee species (Danforth *et al*. 2006; Ascher & Pickering 2015), the western honeybee (*Apis mellifera* L.) is considered the most important commercial pollinator (Potts *et al*. 2010), although seed, nut and fruit set of many pollinator‐dependent crop plants also increase with visitation by wild bees, independent of honeybee density (Garibaldi *et al*. 2013), including on small tropical farms (Garibaldi *et al*. 2016). Recent evidence suggests that a small subset of bee species may fulfil most pollination of crop plants (Kleijn *et al*. 2015; Winfree *et al*. 2015).

In recent years an alarming decline of pollinators has been reported (Potts *et al*. 2010; Bartomeus *et al*. 2013), threatening the ecosystem service of pollination (Cardinale *et al*. 2012). Drivers of bee decline are diverse and include: large‐scale clearance of habitat and habitat deterioration (e.g. through reduced floral availability), pesticide misuse, emergent pathogens and climate change (Potts *et al*. 2010; Roulston & Goodell 2011; Scheper *et al*. 2013), as well as potential interactions among these factors (Vanbergen 2013). Habitat clearance and deterioration in particular are major ongoing concerns (Crowther *et al*. 2015; Ghazoul *et al*. 2015; van Straaten *et al*. 2015). They are considered the most important drivers of biodiversity loss in general (Newbold *et al*. 2015) and of bee decline in particular (Brown & Paxton 2009; Winfree *et al*. 2009).

Studies investigating pollinator decline at localities with extreme habitat loss have shown that increasing intensification of agriculture is correlated with a decline in the diversity of pollinators, principally wild bees (review in Winfree *et al*. 2009). However, less is known about the effects of moderate habitat loss on bee diversity and pollination services provided by them, especially in the tropics, where agricultural production is increasingly pollinator dependent (Klein, Steffan‐Dewenter & Tscharntke 2003; Aizen *et al*. 2008). In three tropical countries, Indonesia, India and Costa Rica, decreases in forest area have been associated with a drop in pollinator diversity and a decrease in fruit production (Klein, Steffan‐Dewenter & Tscharntke 2003; Ricketts 2004; Brosi, Daily & Ehrlich 2007), suggesting that the amount and proximity of natural habitat to cropland can enhance pollinator abundance and richness (Ricketts *et al*. 2008; Boreux *et al*. 2013; Romero & Quezada‐Euan 2013; Freitas *et al*. 2014).

Tropical subsistence agriculture is often practiced although cutting and burning of trees in what is commonly termed slash‐and‐burn. In Mesoamerica (Mexico and Central America), a type of rotational slash‐and‐burn agriculture, locally known as *milpa*, is the traditional low‐intensity practice in which staples of maize, beans and squashes are produced (Toledo *et al*. 2003). In the northern Yucatán Peninsula, *milpa* maintains a mosaic of managed forest and fallow land and leads to an increase in landscape diversity due to the use of multi‐stage and successional pathways in which native secondary growth vegetation is an integral part of the agricultural system. *Milpa* has been suggested to permit the maintenance of biodiversity and sustainable use of natural resources (Gómez‐Pompa & Kaus 1999; Diemont *et al*. 2011). However, the impacts of such traditional agricultural practices for the pollinator community and pollination services to crops have yet to be evaluated.

Mesoamerica is considered an important biodiversity hotspot (Brooks *et al*. 2002), but has suffered severe loss (>70%) of its original vegetation in the last 30 years (Laurance, Sayer & Cassman 2013). *Milpa* agriculture has allowed maintenance of a large percentage of secondary and original forest cover (80%), with annual deforestation rates for 1990–2006 on the scale of 0·09% (Ramírez‐Delgado, Christman & Schmooka 2015). Whether this system of traditional agriculture, with this degree of deforestation across the Yucatán Peninsula, has an impact on pollination services and crop productivity remains an open question.

One of the most important cash crops in the Yucatán Peninsula is Habanero chilli (*Capsicum chinense* Jacq.); the region produces more than 2500 t of chilli with an annual value of 203 000 USD (SIAP 2013). Chilli is a pollinator‐dependent crop (Cauich *et al*. 2006) and in the Yucatán Peninsula its flowers are visited by a number of bee species, including native *Augochloropsis* spp., *Exomalopsis* spp., *Nannotrigona perilampoides* Cresson, 1878*, Frieseomelitta nigra* Cresson, 1878*, Lasioglossum* spp. and the introduced honeybee, *A. mellifera* (Palma *et al*. 2008). Chilli cultivation has even been suggested to help maintain a diverse and abundant pollinator assemblage in the Neotropics (Macias‐Macias *et al*. 2009). However, which bee species or assemblage of floral visitors plays the most important role in pollinating chilli in the field is not known.

Growing of cash crops like chilli could, paradoxically, reduce pollination service provision (*PSP*) to those same crops, a problem that besets pollinator‐dependent agriculture world‐wide (Kremen, Williams & Thorp 2002; Aizen *et al*. 2008). Using sites in the Yucatán Peninsula of Mexico, we firstly hypothesized that traditional *milpa* agricultural practices and the cropped land impact negatively on bee pollinator communities. Secondly, we hypothesized that negative impacts, if evident, would compromise the pollination of chilli in *milpa* agriculture. Our third hypothesis was that pollination services to chilli are provided by a wide range of bee species; answering this hypothesis helped us resolve differences in our responses to the first two hypotheses.

## Materials and methods

The Yucatán Peninsula is characterized by a low‐altitude landscape consisting of a relatively heterogeneous matrix of agricultural, suburban and early successional habitats (c. 20% land cover) within natural tropical forest (c. 80% land cover) (Ramírez‐Delgado, Christman & Schmooka 2015). Traditional *milpa* is the usual agricultural practice; forest is cut and burned and the resulting small fields are used for the cultivation of staples (maize and beans), cash crops (chilli, tomato, melon) and various other non‐market plants for 2–5 years before being abandoned, after which secondary forest re‐establishes (Diemont *et al*. 2011). All of our sites were subjected to this agricultural practice.

### Experimental design

**Figure 1:**
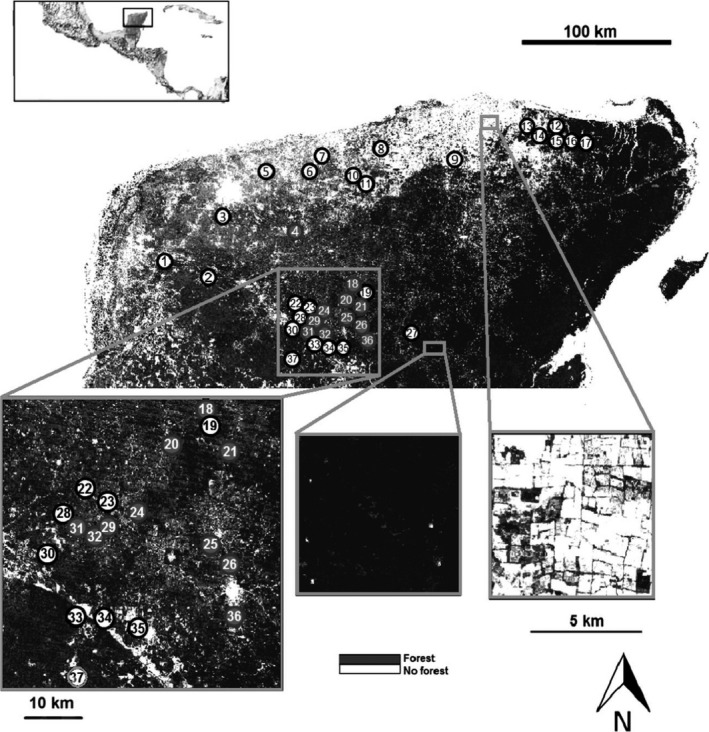
Location of the 37 study sites (numbered 1–37) in the Yucatán Peninsula of Mexico (insert locates the Yucatán Peninsula in Mexico and Central America). Dark circles represent sites where chilli pollination experiments were performed. The open white area is the major city of Mérida. Inserted are two high‐resolution close‐ups of forest‐rich and crop‐rich areas. A key to site names is provided in Table S1.

We selected 37 sites in the Yucatán Peninsula distributed across a gradient of forest loss (45% forest varied from 0% to 98% across sites) at which chilli was grown by local peasant farmers (Fig. 1, Table S1, Supporting Information). Distances between sites were >2 km (mean: 42·8 km; range 2·8–244·6 km; see Fig. 1), beyond the foraging range of most bee species sampled (mean thorax width: 2·07 mm, range 0·77–3·50 mm; estimated mean foraging range, 1·75 km, range 0·30–3·10 km, Gathmann & Tscharntke 2002; Greenleaf *et al*. 2007). We therefore considered bee communities at different sampling sites to be independent.

### Land cover and scale determination

We quantified the environment surrounding each site by estimating the percentage of three specific land cover types: (i) forest, natural vegetation comprising trees with a height >10 m (*Forest*); (ii) fallow land, traditional domestic gardens surrounding dwellings, and pastures (*FGP*) which are an integral component of the *milpa* agricultural system; and (iii) cropland, mixed cropping systems at a distance from permanent settlements and with traditional and culturally important maize, beans and Habanero chilli (all annuals) with some citrus and avocado (perennials) (*Crops*).

Land use was estimated using GE Grids tool (Jacobson *et al*. 2015), which creates an interactive user‐specified grid laid over high‐resolution images from Google Earth^®^ V6.2.2. We used a 40 × 40 grid cell square at a resolution of 50 m (total area of 4 km^2^) from the centre of each of our sampling sites using images from 2010 to 2011 (accessed: April–May 2016). Each grid cell was scored for the percentage (±5%) of *Crops*,* FGP* and *Forest* (for methodological details, see Jacobson *et al*. 2013). We recorded land cover at increasing radii of 200, 300, 400, 500, 600, 700, 800, 900 and 1000 m from the centre of each sampling site (chilli field) within each grid.

We then calculated a landscape diversity index (H*s*) per radius (see Murray *et al*. 2012), which we term the Land‐cover diversity index (*Lc‐diversity*). To estimate the spatial scale at which bee communities responded to surrounding land use, we then correlated landscape composition and H*s* with species richness and abundance of bees using Spearman rank correlation coefficients. Both total bee species richness and abundance showed the highest correlation coefficient with land cover at or near the 300‐m scale (one of eight values were maximal at the 700‐m scale and seven of eight at the 300‐m scale; Table S2). Thus, the 300‐m distance was used in further analyses.

### Bee community sampling

At each site, abundance and species richness of the bee community visiting chilli flowers was quantified on 1–2 days during peak chilli bloom. Across all sites, we sampled the bee community in chilli‐growing areas for at least 1 day every week from May to June in 2010 and from May to August in 2011 (see Table S1). Fifteen yellow and 15 blue pan traps filled with unscented soapy water were employed at every site, placed 10 m apart and at a height of 50 cm (Tuell & Isaacs 2009), the approximate height of chilli flowers, to sample flying insects potentially visiting chilli flowers. To sample flower visitors directly, transects of 250 m were walked through the middle of the crop for 15 min by an observer carrying an insect net, and all visitors to chilli flowers within a width of 2·5 m were collected. Transect walks were conducted four times per site, at 08:00, 10:00, 12:00 and 14:00, for a total of 1 h per site.

As the majority of visitors to chilli flowers were bees, we limited our identification of pan trap and transect walk material to this taxon. The non‐native honeybee was rarely recorded in chilli fields (four bees across all the sites, collected using pan traps, representing <0·2% of all visitors), despite being common in the Yucatán Peninsula (Moritz *et al*. 2013). All sampled wild bees were determined to species or morphospecies by RA and HM‐V using a reference collection housed at the Autonomous University of Yucatán. Sample‐based accumulation curves implemented in PAST v3.06 (Hammer, Harper & Ryan 2001) indicate that our sampling effort captured a large proportion of the species diversity present at each site (Fig. S1). We used the Chao‐1 richness estimator to evaluate bee species richness within sites, as implemented in PAST v3.06 (Hammer, Harper & Ryan 2001). Bee community composition differed between sampling methods (mean Jaccard index = 0·25 ± 0·09, Fig. S2). Yet bee species richness obtained using pan traps and transect walks was highly correlated (Spearman's rank correlation rho = 0·85, *P *<* *0·01, Fig. S3), as was the abundance of bees derived from pan traps vs. transect walks (rho = 0·29, *P *=* *0·04, Fig. S4). The two methods of insect collection were therefore similarly efficient, although each collected different components of the flying insect community. Therefore, when testing the relationship between bee species richness and land use, we used a data set in which our pan trap and transect walk data were combined as they were complementary. However, as pan trapping may be biased, for example, in the selective capture of bee species, and as transect sampling is potentially biased by the experience of the collector (Westphal *et al*. 2008), we also analysed separately data derived from pan trapping and transect walks when testing the relationships between chilli pollination, bee community and land use.

### Statistical analysis of the bee community and environmental variables

Spatial autocorrelation of bee communities across sites was tested using two approaches. Firstly, we used Moran's I to test for spatial autocorrelation of both abundance and species richness. Secondly, we used a Mantel test to correlate geographic distances between sites against the abundance‐based Jaccard similarity index of bee communities between sites using EstimateS v. 9.1.0 (Colwell *et al*. 2012).

We then employed Canonical Correspondence Analysis (CCA) within the package ‘vegan’ v. 2.2‐1 (Oksanen *et al*. 2016) in R v. 3.1.0 (R Development Core Team 2014) to investigate the relationship between bee community composition and surrounding land cover (*Forest, FGP, Crop*s and *Lc‐diversity* index) at the 300‐m scale. Data from pan traps and transect walks using the 700‐m scale gave similar results (results not shown). We subsequently used Spearman rank correlations to determine relationships between landscape variables (*Forest*,* FGP*,* Crops, Lc‐diversity*) and groups of bees: stingless bees, nonstingless bees, cavity nesters and ground nester. Additionally, we used a general linear model (GLM; R Development Core Team 2014) to test for the effect of landscape composition on Chao‐1 estimates of bee abundance and richness derived from either transect walks, pan traps or both data sets combined.

### Pollination service provision

A pollination experiment was conducted at 11 of the 37 sites, all localized in the centre of the Yucatán Peninsula (Fig. 1), to quantify the ecosystem service of pollination to chilli. Although we did not run pollination experiments at sites in the north of the Yucatán Peninsula, the percentage of forest was similar in both areas (centre: mean = 25·9, SE = 5·5; north: mean = 30·8, SE = 7·5; Fig. S5) and differences in bee community composition between northern and central sites (Jaccard index of dissimilarity = 0·20; Fig. S6) were similar to differences in bee community composition among northern sites (Jaccard = 0·25) or among central sites (Jaccard = 0·27). We therefore consider our analysis of pollination at sites in the centre of the Yucatán Peninsula to be likely representative of the whole peninsula.

At these 11 sites, chilli was grown commercially as a cash crop using a conventional Yucatecan‐managed cropping system in which insecticides and fertilizers were applied at least nine times during the 90‐day growing season of chilli. During the peak of bloom in May and June 2011, five plants were randomly selected at each site and, for each plant, three randomly selected flowers were exposed to one of three treatments: cross‐pollination (treatment H: flowers hand cross‐pollinated then bagged with a 1‐mm mesh insect netting), open pollination (treatment O: flowers left unbagged the entire time), and zero insect pollination (treatment B: flowers permanently bagged with a 1‐mm mesh insect netting, allowing only wind and very small insect pollination). After 45 days, chilli peppers (fruits) were harvested, weighed and seeds per pepper counted. For the calculation of seed set and fruit weight, all failed flowers in each treatment were either included or excluded in separate analyses. As the data could not be fitted to a statistical distribution, a nonparametric Kruskal–Wallis test was used to test for differences between the three pollination treatments: H, O and B; followed by Mann–Whitney–Wilcoxon tests to differentiate between treatments H and O.

We then calculated *PSP* for each site using an index we adapted from Spears’ (1983) single‐visit pollination efficiency index, in which:

PSP=(O−B)/(H−B),

where *H*,* O* and *B* are seed set or fruit weight for their respective treatments. The *PSP* index ranges from 0 (no‐service provision) to 1 (high‐service provision) and represents our measure of the ecosystem service of pollination to chilli. To determine pollination shortfall across sites, we used fruit weight; results did not differ when using seed set.

To test the effects of land cover on bee communities (abundance and species richness from pan traps, transect walks or both methods combined) and *PSP*, and the effects of bee communities on *PSP*, we used linear models (LMs). To avoid overfitting, we excluded land cover variables that were highly correlated. *Lc‐diversity* was positively correlated with *Crops* (rho = 0·75, *P *>* *0·01), and *Forest* was negatively correlated with *FGP* (rho = −0·69, *P < *0·01), whereas *FGP* was not correlated with either *Crops* (rho = −0·21, *P *=* *0·41) or *Lc‐diversity* (rho = −0·14, *P *=* *0·51) (Table S3). Hence, we used *FGP* and *Crops* as land cover variables in our analyses (but we also tested models including *Forest*). To generate comparable estimates, all variables were standardized to a mean of zero and a standard deviation of one. All LM assumptions were checked visually and models were simplified by backward stepwise selection based on the Akaike Information Criterion (AIC). Analyses were performed in the R statistical software (R Development Core Team 2014).

Additionally, we used structural equation modelling (SEM) in AMOS v. 7.0 (Arbuckle 2006) to explore causal relationships between land cover, pollinators and pollination by disentangling the direct and indirect pathways linking land cover, bee richness and bee abundance (from pan trapping and transect walks analysed together or separately), *Lasioglossum* sp. 1 abundance and *PSP*. Starting with a complete model (all hypothesized effects), we selected the best model by removing the non‐informative paths (low standardized coefficient and non‐significant *P*‐values) using χ^2^‐tests, AIC, and root mean square error of approximation model fit indices (see Table S4) (Kline 2011).

## Results

### Bee communities

A total of 2215 bee specimens, representing 91 morphospecies, were collected in chilli fields across the 37 sites in pan traps and on transect walks (Table S5). Most individuals and species belonged to the family Apidae (44 spp.), followed by Halictidae (21 spp.), Megachilidae (18 spp.), Colletidae (5 spp.) and Andrenidae (3 spp.) (Fig. S7). An undescribed sweat bee (*Lasioglossum* sp. 1) was the most abundant species (Table S5) and represented 22% of all individuals collected (37% of pan trap specimens and 8% of transect walk specimens). Three other bee species, all members of the family Apidae, were also abundant: the solitary bees *Melissodes tepaneca* Cresson, 1878 (13%) and *Ceratina* sp. 1 (9%), and the eusocial stingless bee *Trigona fulviventris* Guérin‐Méneville, 1845 (c. 5%).

There was no evidence of spatial autocorrelation in bee abundance (Moran's *I *=* *−0·02, *P *=* *0·85), species richness (Chao‐1) (Moran's *I *=* *−0·04, *P *=* *0·82) or the Jaccard index of community similarity (Mantel test, rho = −0·04, *P *=* *0·73) across sites. We therefore consider our measures of bee abundance and richness to be independent across sites.

### Land cover as a driver of the bee community

Across sites, land cover at the 300‐m scale was dominated by *FGP* (45%), while the area covered by *Forest* and that under *Crops* represented 29% and 26% of the surrounding landscape respectively. Our CCA revealed that the area of *FGP* had a significant effect on bee community composition (*F* = 1·68, *P *=* *0·015, 14% constrained variance; Table S6 and Fig. S8), showing that an increase in the area of *FGP* is associated with a marked change in bee species composition.

**Figure 2:**
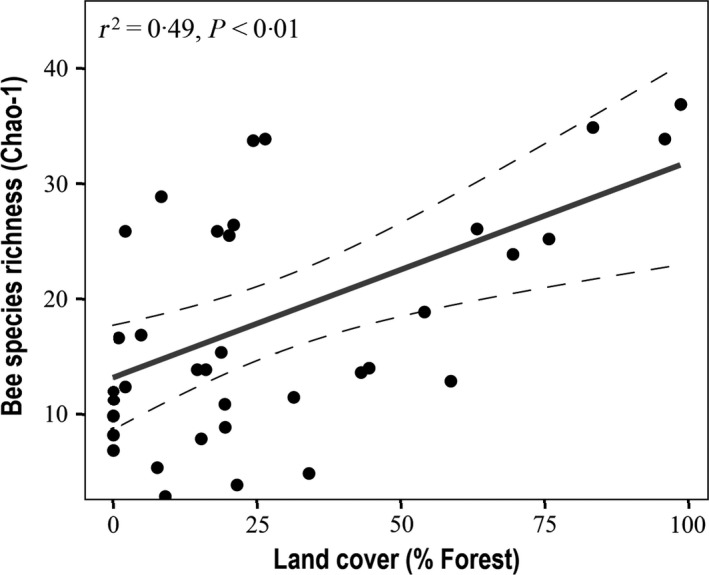
Relationship between bee species richness and the percentage of forest for all 37 sampling sites. Areas under the dashed lines indicate 95% confidence intervals.

Using data from both pan traps and transect walks, we found that sites with greater forest cover harboured a far greater diversity of bee species (Spearman rho = 0·50, *P = *0·02, Table S7 and GLM *Z*._36_ = 7·01, *P *<* *0·01; Table S8, Fig. 2). Sites with greater forest cover also harboured significantly higher bee abundance (GLM *Z*._36_ = 5·54, *P *<* *0·01, Table S8), except for ground‐nesting species, which tended to decrease with forest cover (rho = −0·22, *P = *0·09) or to increase with the area of *FGP* (rho = 0·20, *P = *0·09, Table S7). In agreement with these results, bee species richness and abundance decreased with the area of *Crops* at each site (richness: GLM *Z*._36_ = −4·23, *P *<* *0·01; abundance: GLM *Z*._36_ = −1·59, *P *=* *0·11; Table S8), reflecting a negative impact of agriculture on pollinator communities.

Landscape composition was not significantly related to the richness or abundance of cavity‐nesting bees (*Forest*; rho = 0·14, *P *=* *0·30, *FGP*; rho = 0·10, *P *=* *0·45; *Crops*; rho = 0·03, *P *=* *0·81; Table S7). Total richness and abundance of bee species were little impacted by the area of land covered by *FGP* (Tables S7 and S8). In contrast, the ground‐nesting species *Lasioglossum* sp. 1 (rho = 0·40, *P *=* *0·02), *M. tepaneca* (rho = 0·30, *P = *0·06) and *T. fulviventris* (rho = 0·24, *P *=* *0·09) increased in abundance with *FGP* (Table S7). Results were qualitatively the same when abundance and richness from pan traps or transect walks were used separately (Table S8).

### Pollination

Across all sites, bagged flowers (treatment B) either failed or set very small fruits with few seeds (Table 1). Wind pollination and small insects therefore seemed to play little or no role in Habanero chilli pollination at our sites. Using data that include failed flowers, unbagged flowers (treatment O), representing pollination provided by insects, had good seed set (mean = 23·0, SE = 1·3 seeds per fruit) and fruit weight (mean = 5·27, SE = 0·27 g), but hand cross‐pollinated flowers (treatment H) set the most seeds (mean = 30·2, SE = 1·2 seeds) and produced the heaviest fruits (mean = 6·62, SE = 0·23 g), differing significantly from the other two treatment (Kruskal–Wallis tests: seeds set per fruit, *W* = 7·75, *P *<* *0·01; fruit weight, *W* = 7·56, *P *<* *0·01) and from the Open treatment (Mann–Whitney–Wilcoxon: seeds set: *Z* = −3·9247, *P *<* *0·01; fruit weight: *Z* = −4·71, *P *<* *0·01). Analyses excluding the failed flowers gave similar results (Table 1).

**Table 1:** Mean seed set and fruit weight for experimental chilli pollination treatments across 11 sites (dark circles in Fig. 1)

Treatment (*n* = 165)	Seeds per fruit				Fruit weight (g)				
	Mean (including failed)	SE	Mean (excluding failed)	SE	Mean (including failed)	SE	Mean (excluding failed)	SE	% failed
Hand cross‐pollination (H)	30·20†	1·22	36·61†	0·76	6·62†	0·23	8·07†	0·07	18
Open pollination (O)	23·01¶	1·32	33·73¶	1·02	5·27¶	0·27	7·73¶	0·08	32
Continually bagged (B)	3·05§	0·58	22·00§	1·39	0·96§	0·17	6·93§	1·39	86

Symbols within the same column represent significant differences (Kruskal–Wallis test with *a posteriori* comparison of means, *P *<* *0·05).

Pollination Service Provision varied between 0·45 and 1·23 across sites, calculated as number of seeds per flower, and between 0·45 and 1·06, calculated as fruit weight per flower (Table S9; values >1 likely arise from biological variation or experimentally induced damage during bagging of flowers in treatment H). *PSP* measured as number of seeds per fruit was highly correlated with *PSP* measured as fruit weight (*R* ^2^ = 0·91, *P *<* *0·01). Hence, we present only the results from *PSP* measured as fruit weight. Results did not differ when *PSP* was estimated as number of seeds per fruit or when values of *PSP* >1 were rounded down to 1.

### Relationships between bees, land cover and *PSP*

**Figure 3:**
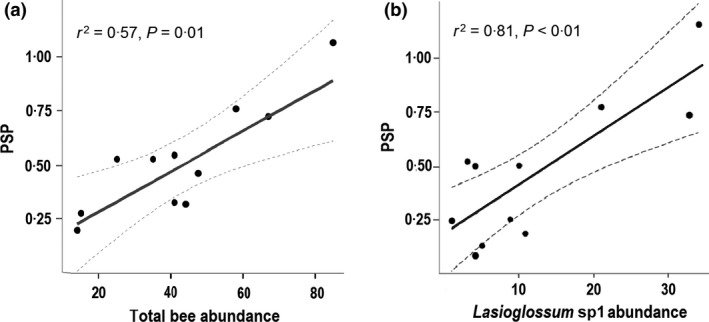
Relationship between pollination service provision (*PSP*) and bee abundance (derived from pan traps) at 11 experimental sites: (a) total bee abundance; (b) abundance of *Lasioglossum* sp. 1 (areas under the dashed lines indicate 95% confidence intervals).

Using LMs, we found a significant positive relationship between *PSP* and bee abundance across sites (*t*_8_ = 3·42, *P *<* *0·01; Fig. 3a), but a non‐significant negative trend with bee species richness (*t*_8_ = −0·67, *P *=* *0·52; Table S10). We found similar patterns when separately analysing pan trap and transect data sets. The relationship between *PSP* and bee abundance was driven largely by the abundance of *Lasioglossum* sp. 1, with which *PSP* was also significantly positively related (combined data set; *t*_8_ **=* *3·34, *P *=* *0·02; Fig. 3b; we found the same result when separately analysing pan trap or transect walk data; see Table S11). The abundances of the other relatively common bee species were not significantly related to *PSP*:* M. tepaneca* (*t*_8_ = −0·40, *P *=* *0·71), *Ceratina* sp. 1 (*t*_8_ **=−1·26, *P *=* *0·25) and *T. fulviventris* (*t*_8_ = −0·64, *P *=* *0·55) (see also Table S11). *PSP* was also not related to any landscape variable (*Crops: t*_8_ < 0·01, *P *=* *0·99; *FGP*:* t*_8_ < 1·46, *P *=* *0·18; *Forest*:* t*_8_ = −1·48, *P *=* *0·17; Table S12).

**Figure 4:**
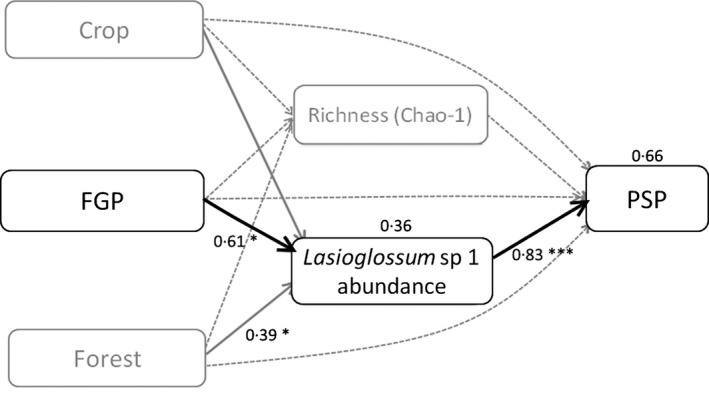
Path diagram from a structural equation model (SEM) to determine the influence of land cover and the bee community on the ecosystem service of pollination (*PSP*) provided to chilli. Squares represent measured variables, straight arrows represent direct causal pathways and curved double‐headed arrows indicate correlations. Explained variation in response variables (bee abundance and *PSP*) is shown above their corresponding squares. Continuous lines represent significant effects whil dotted lines represent non‐significant effects. The values next to arrows represent standardized regression weights. Arrow widths are scaled to standardized path coefficients. The dotted grey lines are non‐significant hypothesized links from the full model. Significance of effects; *P <* *0·05, ***P <* *0·001.

Model selection in our SEM analysis yielded one final path model relating landscape and bee community metrics to *PSP*, with stable fit to our data (Table S4). Overall, SEM analysis (Fig. 4) supported the findings of the LMs (Table S10); there was a significant effect of *Lasioglossum* sp. 1 abundance (pan trap data, but not transect data) on *PSP* (std. coef. = 0·83, *P *<* *0·01; *R* ^2^ = 0·66), which at the same time was positively affected by the area of *FGP* (std. coef. = 0·61, *P *<* *0·05; *R* ^2^ = 0·36). There was also a marginally positive indirect effect of *FGP* through *Lasioglossum* sp. 1 abundance on *PSP* (*P *=* *0·05). The final SEM did not, however, include bee abundance and richness (from pan traps, transects or combined data), area of crops and area of forest due to substantial reduction in model fit (Table S4).

## Discussion

We found that bee diversity was highest in forested areas and lower in cropped land or that under fallow, even in low‐intensity traditional *milpa* agriculture, supporting our first hypothesis that agriculture has a negative impact on pollinators. Although, surprisingly, chilli pollination was enhanced by surrounding fallow, gardens and pasture, and reduced by surrounding forest cover, rejecting our second hypothesis that service provision would drop with a decline in bee diversity. We also reject our third hypothesis that many bee species contribute to chilli pollination; rather, we found that just a small subset, primarily ground‐nesting *Lasioglossum* sweat bee species, were primarily responsible, and that these were associated with fallow, pasture and gardens rather than forested or cropped areas.

### Bee community composition and its relation to land cover

We found all five families of bee reported for the Neotropics at our sites, a pattern of relative abundance typical for the region (Roubik 1992). As *T. fulviventris* was the only common eusocial bee species, our data suggest that solitary and primitive social bees, but rarely eusocial species, are favoured in this agro‐ecosystem.

Reduced species diversity of bees with agricultural intensification has been reported in northern temperate localities (Kremen, Williams & Thorp 2002; Klein *et al*. 2007; Brown & Paxton 2009). We also observed a negative effect of the area of crops on bee species diversity. These data underpin the view that loss of habitat is the most important driver of bee loss across many terrestrial biotopes (Potts *et al*. 2010; Vanbergen 2013; Ghazoul *et al*. 2015). Our CCA revealed *FGP* as the only landscape variable having a significant impact on bee community composition. Others (Lyver *et al*. 2015; Norfolk, Eichhorn & Gilbert 2016) have found that traditional land management including fallow areas increases species diversity. We found the abundance of *Lasioglossum* spp. and other ground‐nesting species increased with the area of *FGP*. This relationship can best be explained by the ecology of members of the genus *Lasioglossum*, which prefer unshaded areas for nesting (Cane 2001). Localities with an intermediate degree of disturbance with different successional stages, as might be found in our *FGP* land use class, may even promote species diversity (Diemont *et al*. 2011).

Forest is the climax community of much of the Yucatán Peninsula (Ramírez‐Delgado, Christman & Schmooka 2015) and might be expected to harbour high bee species diversity. Although we found that bee communities associated with *FGP* contained large numbers of chilli flower visitors, we nevertheless found bee diversity to be positively related to the amount of forest surrounding a site. The positive effect of the proximity of forest for bee distribution and abundance in the tropics has also been observed in coffee, cashew and *Jatropha curcas* L. crops, where orchards closer to forest fragments had a greater diversity of bees (Cane 2001; Ricketts 2004; Boreux *et al*. 2013; Romero & Quezada‐Euan 2013; Freitas *et al*. 2014).

### Chilli pollination

We found that 21% of unmanipulated chilli flowers failed to set fruit across sites. Current provision of the ecosystem service of pollination (average *PSP* across all sites = 0·72 ± 0·23) for chilli in the Yucatán Peninsula is substantial, although less than maximum. Cauich *et al*. (2006) and Palma *et al*. (2008) found similar percentages of fruit set in experimentally controlled pollination of Habanero chilli, in which buzz pollinators like the stingless bee *N. perilampoides* were employed.

Recent attention has focused on the importance of wild bees for crop pollination (Garibaldi *et al*. 2013, 2016). For chilli in the Yucatán Peninsula, the presence of native bees in crop fields has been shown to increase crop yield and quality (Macias‐Macias *et al*. 2009). Although we did not incorporate pollinator single‐visit efficiency assays into our analyses (e.g. Freitas & Paxton 1998), *Lasioglossum* spp. frequently visit habanero flowers (J. Quezada‐Euán pers. comm.). Our data point to *Lasioglossum* sp. 1 as an important pollinator of chilli in the region, underlining the importance of wild bees in crop pollination (Garibaldi *et al*. 2013, 2016). Yet our data also demonstrate that only a small subset of the bee diversity, in our case sweat bees, plays an important functional role in crop pollination (Kleijn *et al*. 2015; Winfree *et al*. 2015).

The current pollination benefits provided by wild bees suggest constraints to agricultural intensification in the Yucatán Peninsula. If agriculture intensifies and more pollinator‐dependent crops are grown, this may at the same time decrease the diversity and abundance of pollinators and the pollination services they provide. Although crops may act as a source of food for bees by providing pollen and nectar (Ollerton, Winfree & Tarrant 2011), they do not provide essential sites for nesting and reproduction (Cane 2001). However, our study demonstrates that traditional slash‐and‐burn *milpa* agriculture could help to maintain the provision of pollination services, probably through successional restoration processes, which simultaneously increase the availability of floral resources and nesting sites and that could enhance the local richness and abundance of bees, particularly ground‐nesting species. Similarly, long‐term studies in California have shown that the use of native plant hedgerows around crops increased the richness of native bees (Kremen & M'Gonigle 2015). Yet, in our study, cavity‐nesting bees did not show a relationship with *FGP* or to any other type of land use, only with landscape diversity (*Lc‐diversity*), suggesting that many other bees, especially cavity‐nesting species, may not find adequate nesting sites in *milpa* systems.

### Relationships between bees, land cover and pollination service

Both LMs and causal modelling (SEM) revealed a positive effect of bee abundance on the pollination of chilli, possibly driven by the abundance of *Lasioglossum* sp. 1, which exhibited the strongest relationship with chilli pollination. Sweat bees such as *Lasioglossum* spp. mechanically buzz flowers of the Solanaceae (Teppner 2005), which may facilitate pollination (Raw 2000). Some sweat bees also exhibit a preference for nesting in open areas (Cane 2001), as provided in fallow lands as part of traditional *milpa* agriculture. Both phenomena may explain why we observed a close relationship between chilli pollination and *Lasioglossum* sp. 1 abundance.

Changes in land use away from traditional practices could have contrasting effects on the community of bees due to their species‐specific foraging and nesting preferences (Brosi *et al*. 2008; Norfolk, Eichhorn & Gilbert 2016). Sweat bees building fossorial nests could benefit from non‐forested areas like those found in fallow land, gardens and pastures. In turn, several members of the Apidae are eusocial and nest within hollow trees (Michener 1974). These species are negatively affected by a reduction in forest cover (Brosi *et al*. 2008). For chilli crop pollination, current *milpa* agricultural practices seem not to impact negatively on its pollinator communities and provision of pollination, but this may not be the case for other crops that depend more on other taxa for their pollination requirements.

## Conclusions and management recommendations

Our study shows that some native bee species profit from the intermediate disturbance resulting from traditional, low‐intensity *milpa* agriculture. Hence, in Neotropical regions, the maintenance of traditional *milpa* practices may be of advantage for some local pollinator communities (Gómez‐Pompa & Kaus 1999; Diemont *et al*. 2011). However, it is important to highlight that other angiosperms may need different pollinator assemblages compared to those associated with chilli crops; such pollinator communities may only be supported by natural habitat. Therefore, as well as valuing semi‐natural successional stages arising from low‐intensity *milpa* agricultural practices to support ground‐nesting bee species such as *Lasioglossum* spp., there is a need to conserve forest so as to maintain bee diversity and the ecosystem service of pollination that they provide to a potentially wide range of other crops and wild flowers.

## Data accessibility

The data sets used in this manuscript are available from Dryad Digital Repository https://doi.org/10.5061/dryad.jk111 (Landaverde‐González *et al*. 2016).

## Supporting information


**Fig. S1.** Sample‐based accumulation curves of bee species richness.

**Fig. S2.** Distribution of the similarity index Jaccard of bee community composition in relation to sampling method.

**Fig. S3.** Bee abundance across sites in relation to sampling method.

**Fig. S4.** Individual bee species abundance across sites in relation to sampling method.

**Fig. S5.** Comparisons of forest cover at central vs. northern sites.

**Fig. S6.** Bee community similarity at central vs. northern sites.

**Fig. S7.** Bee abundance and richness across sites with combined sampling methods.

**Fig. S8.** Canonical Correspondence Analysis for the relationships between bee communities and land use across sites.

**Table S1.** Details of the abbreviation used of field sites and the sampling dates.

**Table S2.** Relationships between land cover and the bee communities across sites.

**Table S3.** Relationships among land cover variables across sites.

**Table S4.** Statistical fit of the models of pollination success in relation to bee communities and land use across sites.

**Table S5.** Details of bee community composition across sites.

**Table S6.** Canonical correspondence analysis of the relationships between bee communities and land use across sites.

**Table S7.** Relationship between bee taxa and land cover variables.

**Table S8.** Statistical modelling of bee community metrics and land use across sites.

**Table S9.** Experimental estimates of pollination service provision across sites.

**Table S10.** Statistical modelling of bee communities with pollination service provision.

**Table S11.** Statistical modelling of the most abundant bees with pollination service provision.

**Table S12.** Statistical modelling of land use with pollination service provision.
