# Supplementary material for: Sweat bees on hot chillies: provision of pollination services by native bees in traditional slash‐and‐burn agriculture in the Yucatán Peninsula of tropical Mexico
Source: J Appl Ecol. 2017 Jan 27;54(6):1814–24. doi: 10.1111/1365-2664.12860 (PMC5697652; doi:10.1111/1365-2664.12860)

**Figure S1. Sample-based accumulation curves of bee species richness.**

Sample-based accumulation curves scaled by the number of individuals in samples, representing bee species richness collected with pan traps and on transect walks for A) the 16 sites sampled in 2010 and B) the 21 sites sampled in 2011, each site represented by a different colour. A large proportion of the biodiversity was captured by our sampling regime, even if the curves were not fully saturated. Site codes are given in blue (see Table S1).


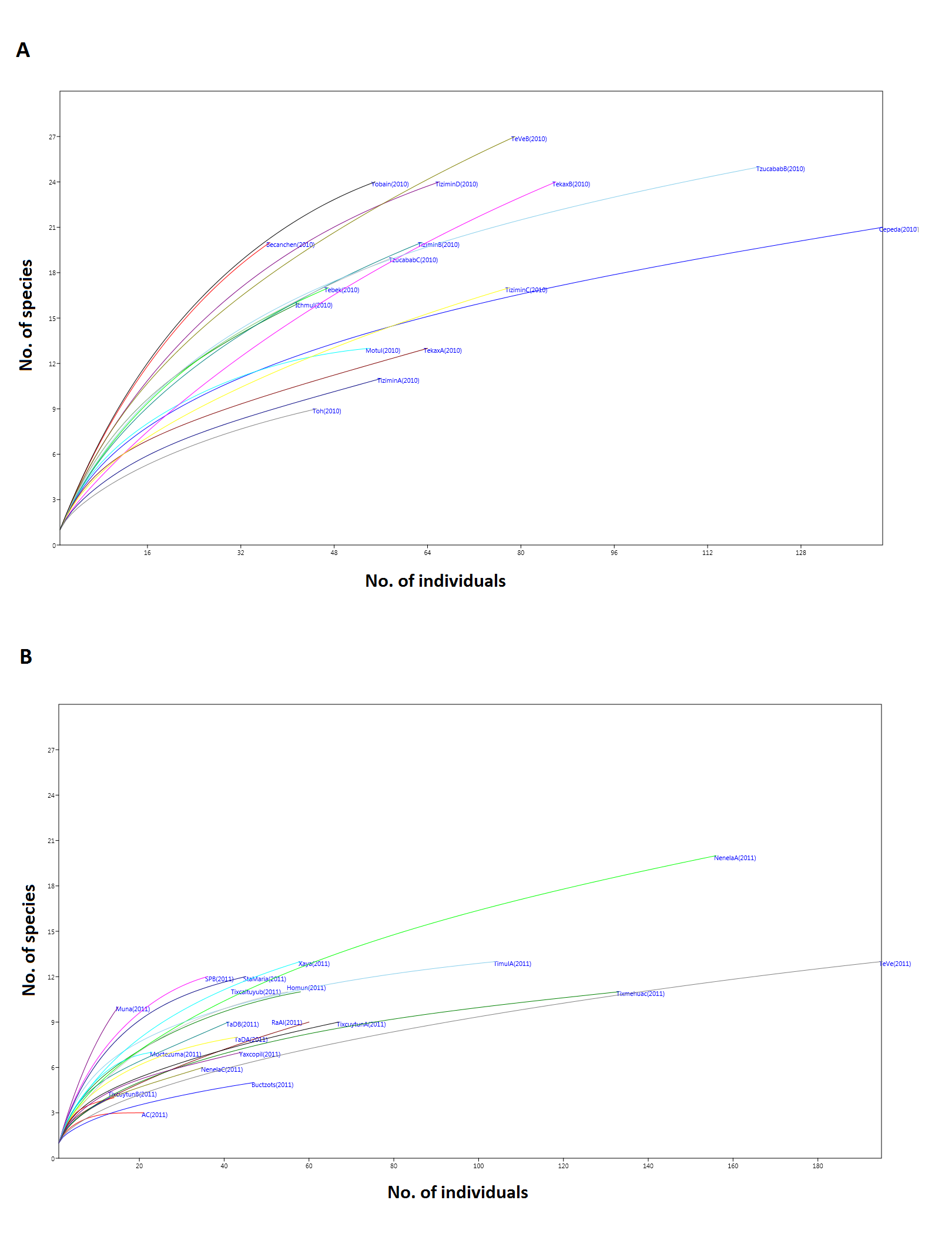

Supplement: Supplementary file 1 — Fig. S1. Sample‐based accumulation curves of bee species richness. [file JPE-54-1814-s001.docx]
