# Supplementary material for: Sweat bees on hot chillies: provision of pollination services by native bees in traditional slash‐and‐burn agriculture in the Yucatán Peninsula of tropical Mexico
Source: J Appl Ecol. 2017 Jan 27;54(6):1814–24. doi: 10.1111/1365-2664.12860 (PMC5697652; doi:10.1111/1365-2664.12860)
Supplement: Supplementary file 2 — Fig. S2. Distribution of the similarity index Jaccard of bee community composition in relation to sampling method. [file JPE-54-1814-s002.docx]

**Figure S2. Distribution of the similarity index Jaccard of bee community composition in relation to sampling method.**

Distribution of the Jaccard index of community similarity (0=totally dissimilar; 1=identical) for the 37 sites, comparing the community composition at each site between methods (pan trapping versus transect walks). The star corresponds to the mean Jaccard index between sampling methods (0.25).

**
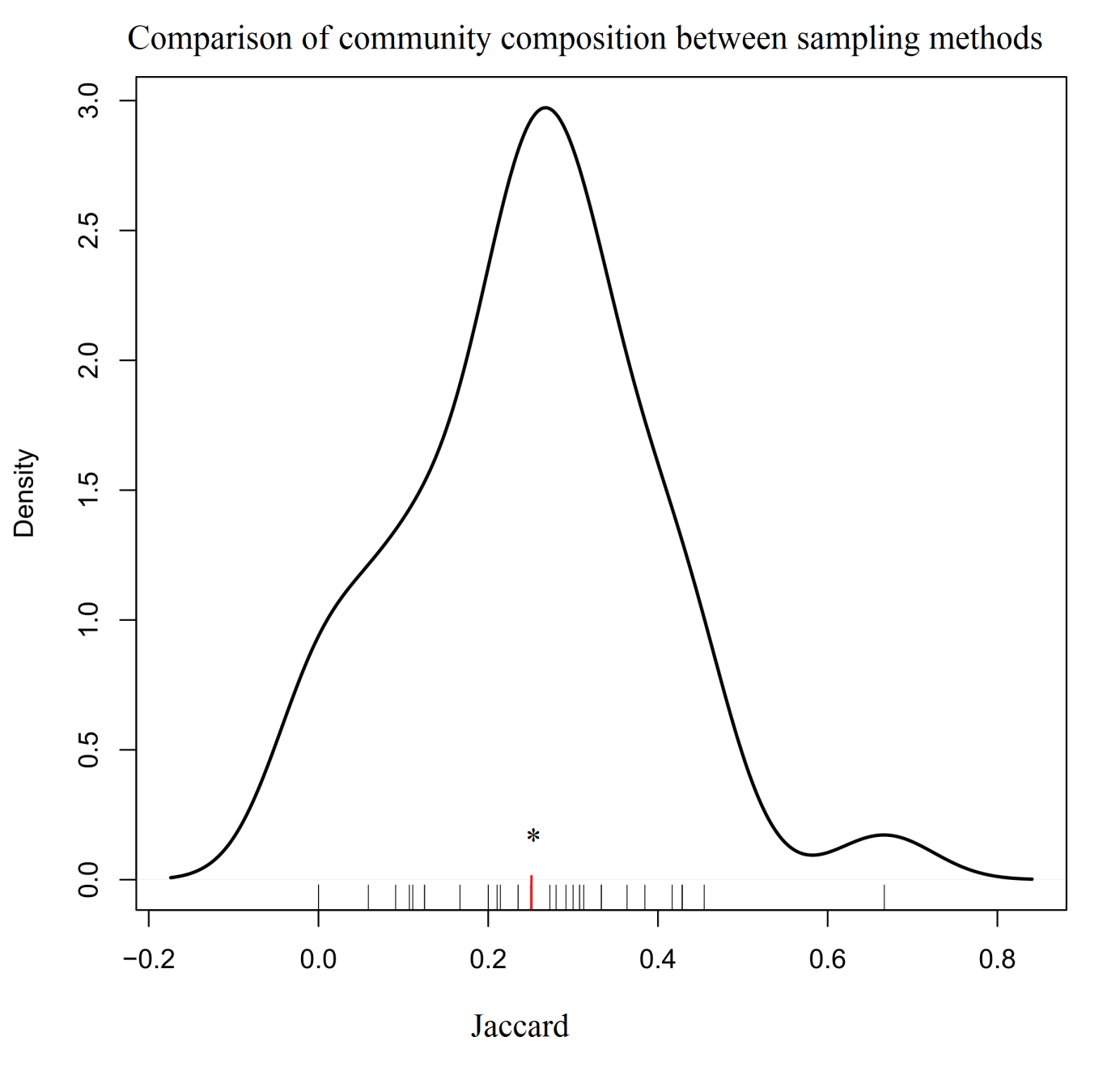
**
