# Supplementary material for: Sweat bees on hot chillies: provision of pollination services by native bees in traditional slash‐and‐burn agriculture in the Yucatán Peninsula of tropical Mexico
Source: J Appl Ecol. 2017 Jan 27;54(6):1814–24. doi: 10.1111/1365-2664.12860 (PMC5697652; doi:10.1111/1365-2664.12860)

**Figure S3. Bee abundance across sites in relation to sampling method.**

Comparison of number of bee species across methods of collection for the 37 sampling sites; a similar number of species was collected by pan trapping (Species pantraps) and on transect walks (Species nets).


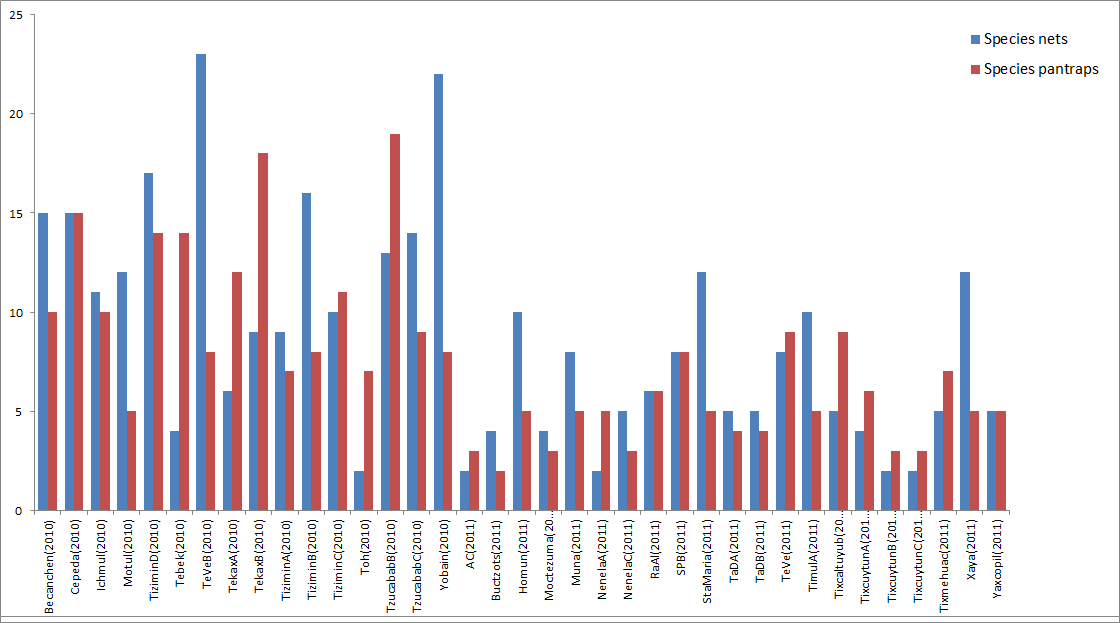

Supplement: Supplementary file 3 — Fig. S3. Bee abundance across sites in relation to sampling method. [file JPE-54-1814-s003.docx]
