# Supplementary material for: Sweat bees on hot chillies: provision of pollination services by native bees in traditional slash‐and‐burn agriculture in the Yucatán Peninsula of tropical Mexico
Source: J Appl Ecol. 2017 Jan 27;54(6):1814–24. doi: 10.1111/1365-2664.12860 (PMC5697652; doi:10.1111/1365-2664.12860)

**Figure S4. Individual bee species abundance across sites in relation to sampling method.**

Comparison of bee abundance across sampling methods (pan trapping versus transect walks) for each of the 91 bee species found in the study. A similar total abundance of bees was collected by pan trapping (Pantraps) and transect walks (Nets), though community composition varied across sampling method.


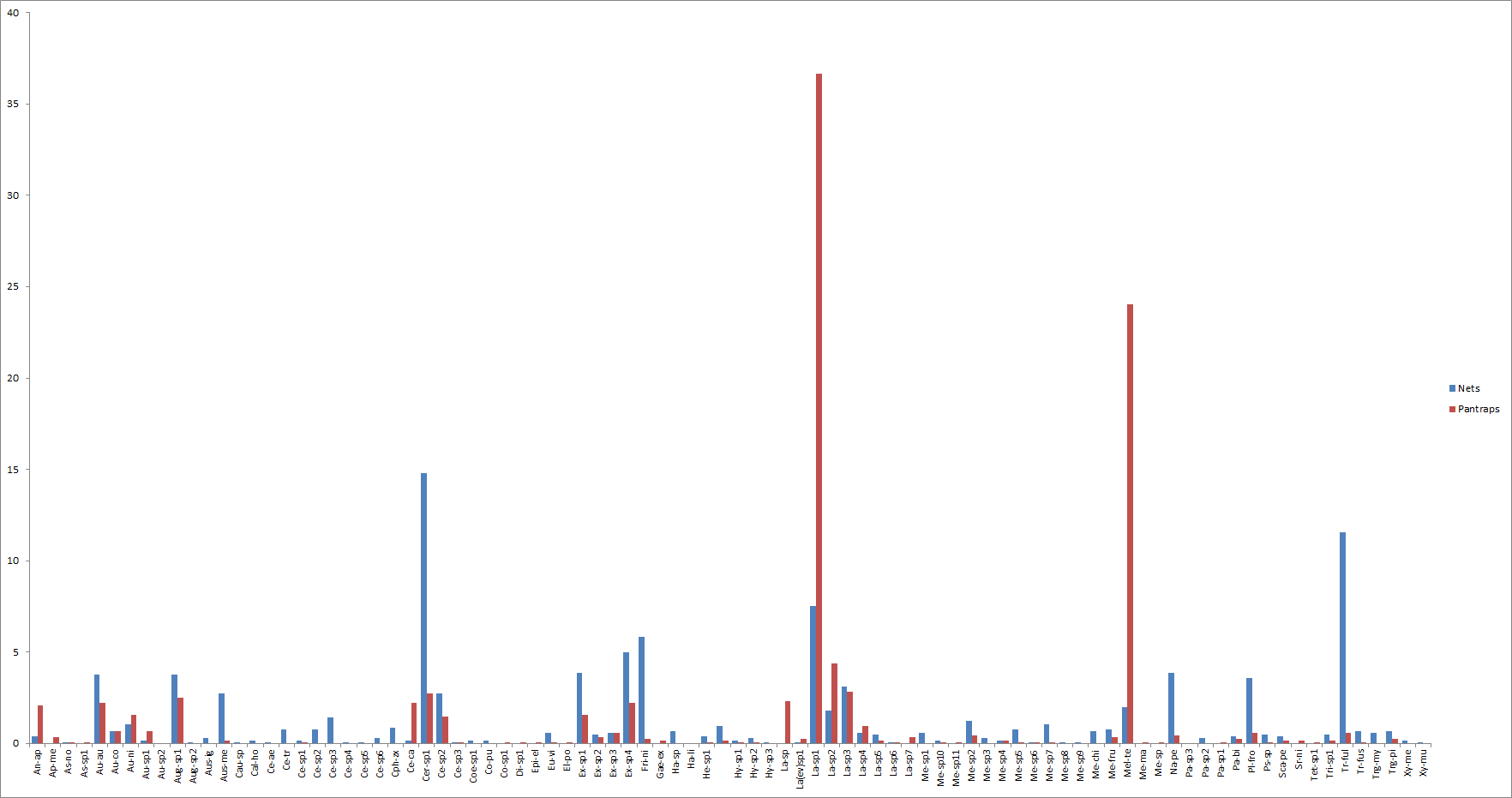

Supplement: Supplementary file 4 — Fig. S4. Individual bee species abundance across sites in relation to sampling method. [file JPE-54-1814-s004.docx]
