# Supplementary material for: Sweat bees on hot chillies: provision of pollination services by native bees in traditional slash‐and‐burn agriculture in the Yucatán Peninsula of tropical Mexico
Source: J Appl Ecol. 2017 Jan 27;54(6):1814–24. doi: 10.1111/1365-2664.12860 (PMC5697652; doi:10.1111/1365-2664.12860)
Supplement: Supplementary file 5 — Fig. S5. Comparisons of forest cover at central vs. northern sites. [file JPE-54-1814-s005.docx]

**Figure S5. Comparisons of forest cover at central *versus* northern sites.**

Mean, standard deviation and CV for the percentage of forest at central and northern sites of the Yucatan Peninsula; central: mean=25.95%, SE=5.53%; northern: mean=30.82%, SE=7.51%.

**
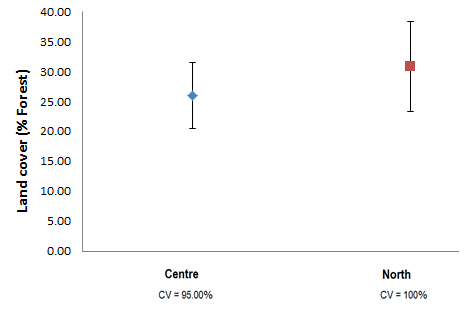
**
