# Supplementary material for: Sweat bees on hot chillies: provision of pollination services by native bees in traditional slash‐and‐burn agriculture in the Yucatán Peninsula of tropical Mexico
Source: J Appl Ecol. 2017 Jan 27;54(6):1814–24. doi: 10.1111/1365-2664.12860 (PMC5697652; doi:10.1111/1365-2664.12860)

**Figure S6. Bee community similarity at central *versus* northern sites.**

Distribution of Jaccard indexes for each pairwise comparison of the bee communities at 37 sites. The letters correspond to the means calculated for a) between northern and central sites (in green), b) within northern sites (in black) and c) within central sites (in red).


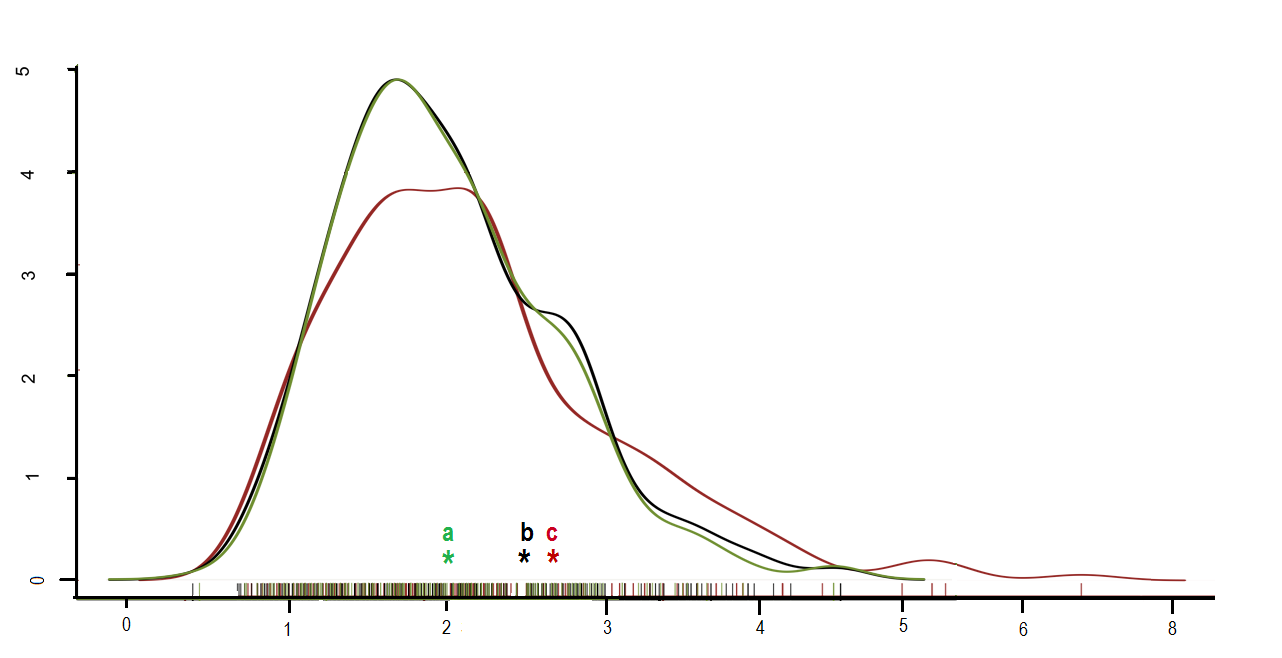

Supplement: Supplementary file 6 — Fig. S6. Bee community similarity at central vs. northern sites. [file JPE-54-1814-s006.docx]
