# Supplementary material for: Sweat bees on hot chillies: provision of pollination services by native bees in traditional slash‐and‐burn agriculture in the Yucatán Peninsula of tropical Mexico
Source: J Appl Ecol. 2017 Jan 27;54(6):1814–24. doi: 10.1111/1365-2664.12860 (PMC5697652; doi:10.1111/1365-2664.12860)

**Figure S7. Bee abundance and richness across sites with combined sampling methods.**

The abundance of each morphospecies collected by pan trapping and transect walks, colour sorted by family. The small graph in the upper left corresponds to the abundance of individuals (dark colour) and richness of species (pale colour) in each family.


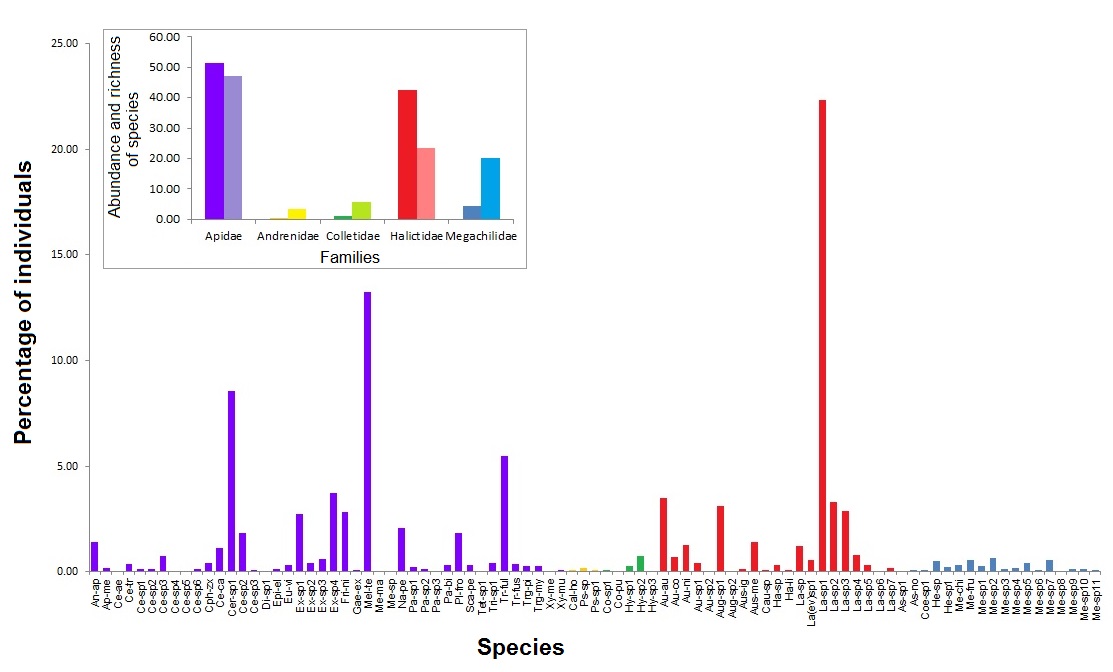

Supplement: Supplementary file 7 — Fig. S7. Bee abundance and richness across sites with combined sampling methods. [file JPE-54-1814-s007.docx]
