# Supplementary material for: Sweat bees on hot chillies: provision of pollination services by native bees in traditional slash‐and‐burn agriculture in the Yucatán Peninsula of tropical Mexico
Source: J Appl Ecol. 2017 Jan 27;54(6):1814–24. doi: 10.1111/1365-2664.12860 (PMC5697652; doi:10.1111/1365-2664.12860)

**Figure S8**. **Canonical Correspondence Analysis for the relationships between bee communities and land use across sites.**

Canonical correspondence analysis (CCA) ordination graph showing the relationship between bee composition and the different land cover classes as explanatory variables. The eigenvalues of axis 1 (CCA1; horizontal) and axis 2 (CCA1; vertical) are 0.3420 and 0.2737, respectively. The proportion of land covered by *FGP* was the only statistically significant factor explaining bee community composition. The dashed red lines represent the direction and strength of the canonical variables, the thick continuous red line indicates the effect of FGP on bee community composition. See supporting information Table S1 for site numbers (black numbers) and Table S5 for bee species codes (blue letters).


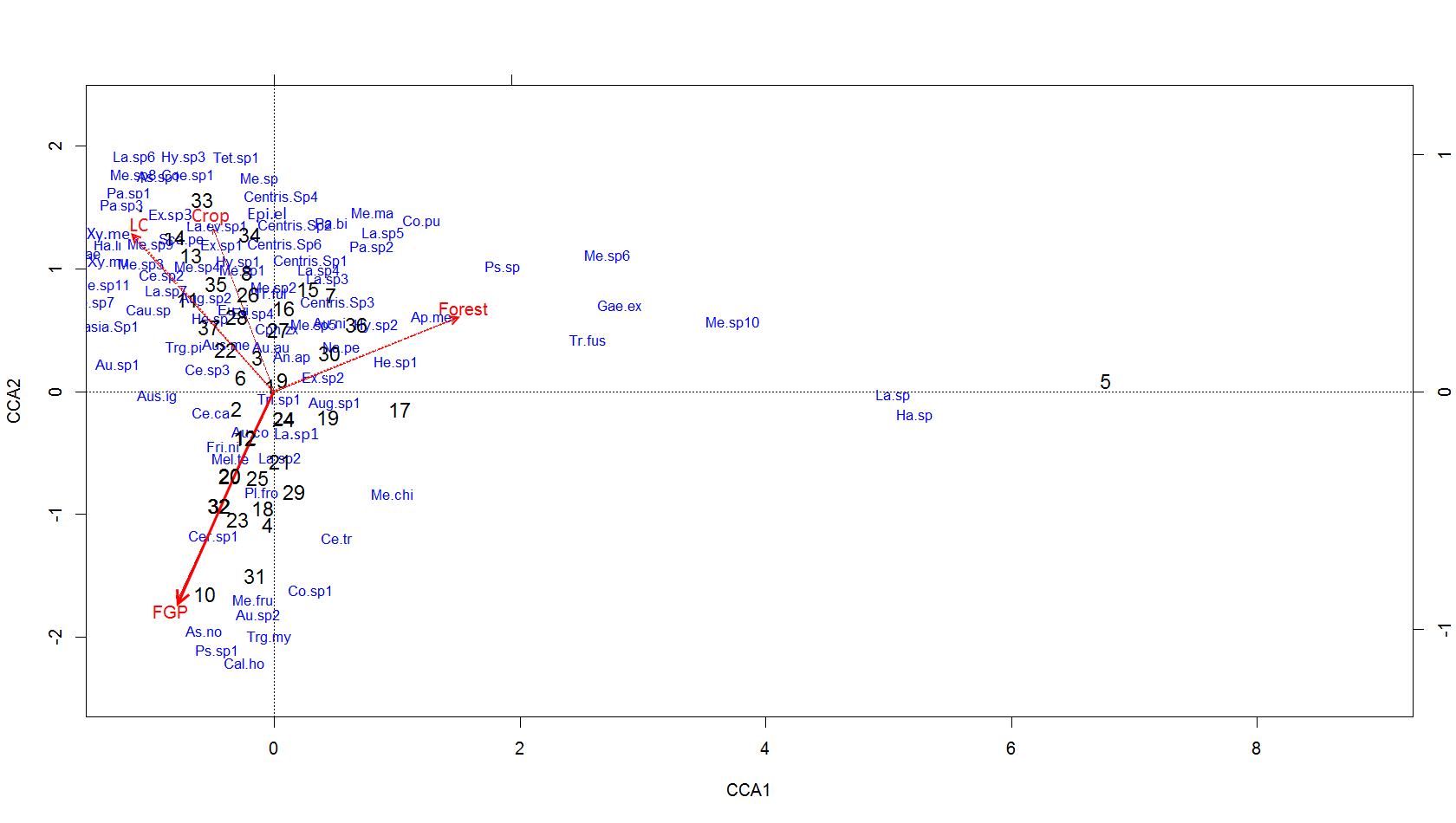

Supplement: Supplementary file 8 — Fig. S8. Canonical Correspondence Analysis for the relationships between bee communities and land use across sites. [file JPE-54-1814-s008.docx]
