# Supplementary material for: Sweat bees on hot chillies: provision of pollination services by native bees in traditional slash‐and‐burn agriculture in the Yucatán Peninsula of tropical Mexico
Source: J Appl Ecol. 2017 Jan 27;54(6):1814–24. doi: 10.1111/1365-2664.12860 (PMC5697652; doi:10.1111/1365-2664.12860)
Supplement: Supplementary file 9 — Table S1. Details of the abbreviation used of field sites and the sampling dates. [file JPE-54-1814-s009.docx]

**Table S1. Details of the abbreviation used of field sites and the sampling dates**

Numbers, names and codes of the field sites in the Yucatan Peninsula at which bees were collected, with sampling date. Sites in which the pollination experiment was performed are highlighted using a pink background. Site numbers and codes correspond to those in Figures 1, S3 and S8.

| **Site number** | **Sites names** | **Sites codes** | **Sampling date** |
| --- | --- | --- | --- |
| 1 | Cepeda | Cepeda(2010) | 19-20/May/2010 |
| 2 | Muna | Muna(2011) | 22/Jul/2011 |
| 3 | Tebek | Tebek(2010) | 4/May/2010 |
| 4 | Homun | Homun(2011) | 28/Jun/2011 |
| 5 | Toh | Toh(2010) | 8-9/May/2010 |
| 6 | Motul | Motul(2010) | 10/Jun/2010 |
| 7 | Yobain | Yobain(2010) | 11/Jun/2010 |
| 8 | Buctzots | Buctzots(2011) | 29/Jul/2011 |
| 9 | TiziminA | TiziminA(2010) | 30/May/2010 |
| 10 | Tekal de Venegas A | TeVe(2011) | 21/May/2011 |
| 11 | Tekal de Venegas B | TeVeB(2010) | 15/Jun/2010 |
| 12 | Moctezuma | Moctezuma(2011) | 30/May/2011 |
| 13 | Rancho Alegre | RaAl(2011) | 1/Jun/2011 |
| 14 | Tizimin D (Santa Maria | TiziminD(2010) | 5/Jun/2010 |
| 15 | Tizimincen C | TiziminC(2010) | 3/Jun/2010 |
| 16 | TiziminB | TiziminB(2010) | 1/Jun/2010 |
| 17 | San Pedro Bacab | SPB(2011) | 31/May/2011 |
| 18 | Tixcaltuyub | Tixcaltuyub(2011) | 24/Jun/2011 |
| 19 | Santa María | StaMaria(2011) | 26/Jun/2011 |
| 20 | Nenela C | NenelaC(2011) | 14/Jun/2011 |
| 21 | Timul A | TimulA(2011) | 09/Jun/2011 |
| 22 | Xaya | Xaya(2011) | 5/Aug/2011 |
| 23 | Tixmehuac | Tixmehuac(2011) | 15/Jun/2011 |
| 24 | Nenela A | NenelaA(2011) | 9/May/2011 |
| 25 | TahDziu B | TaDB(2011) | 18/Jun/2011 |
| 26 | TahDziu A | TaDA(2011) | 6/Jun/2011 |
| 27 | Ichmul | Ichmul(2010) | 31/May/2010 |
| 28 | Tekax A | TekaxA(2010) | 2/May/2010 |
| 29 | Tixcuytun A | TixcuytunA(2011) | 7/Jun/2011 |
| 30 | Tekax B | TekaxB(2010) | 12-13/May/2010 |
| 31 | Tixcuytun B | TixcuytunB(2011) | 17/Jun/2011 |
| 32 | Tixcuytun C | TixcuytunC(2011) | 22/Jun/2011 |
| 33 | Alfonso Caso | AC(2011) | 13/May/2011 |
| 34 | Tzucabab C | TzucababC(2010) | 9/Jun/-2010 |
| 35 | Tzucabab B | TzucababB(2010) | 24/May/2010 |
| 36 | Yaxcopil | Yaxcopil(2011) | 1/Aug/2011 |
| 37 | Becanchen | Becanchen(2010) | 8/Jun/2010 |
