# Supplementary material for: Sweat bees on hot chillies: provision of pollination services by native bees in traditional slash‐and‐burn agriculture in the Yucatán Peninsula of tropical Mexico
Source: J Appl Ecol. 2017 Jan 27;54(6):1814–24. doi: 10.1111/1365-2664.12860 (PMC5697652; doi:10.1111/1365-2664.12860)
Supplement: Supplementary file 10 — Table S2. Relationships between land cover and the bee communities across sites. [file JPE-54-1814-s010.docx]

**Table S2. Relationships between land cover and the bee communities across sites.**

Spearman rank correlation coefficients of the relationship between either total bee abundance or bee species richness (combined pan trap and transect walk data) and land cover at different spatial scales from a site’s centre. Land cover is the proportion of land covered by: agricultural fallow land, home gardens and pasture (*FGP*); primary or secondary growth forest (*Forest*); cropland (*Crops*), comprising staples (maize, beans), cash crops (e.g. chilli) and orchards; and an overall index of the diversity of land cover (*Lc-diversity*) of all three land cover classes: *FGP*, *Forest* and *Crops*. The largest absolute correlation coefficient (positive or negative) of a row is given in bold.

| ***FGP*** | **Distance class from the centre of a site (in m)** | | | | | | | |  |
| --- | --- | --- | --- | --- | --- | --- | --- | --- | --- |
| **Distance** | 200 | 300 | 400 | 500 | 600 | 700 | 800 | 900 | 1000 |
| **Abundance** | 0.04 | **0.11** | 0.09 | 0.11 | 0.09 | 0.07 | 0.06 | 0.05 | 0.06 |
| **Richness (Chao-1)** | -0.31 | -0.28 | -0.27 | -0.28 | -0.30 | **-0.32** | -0.31 | -0.32 | -0.30 |

| ***Forest*** | **Distance class from the centre of a site (in m)** | | | | | | | |  |
| --- | --- | --- | --- | --- | --- | --- | --- | --- | --- |
| **Distance** | 200 | 300 | 400 | 500 | 600 | 700 | 800 | 900 | 1000 |
| **Abundance** | -0.25 | **-0.26** | -0.20 | -0.13 | -0.06 | -0.01 | 0.03 | 0.03 | 0.04 |
| **Richness (Chao-1)** | 0.05 | **0.23** | 0.12 | 0.16 | 0.19 | 0.21 | 0.22 | 0.21 | 0.18 |

| ***Crop*** | **Distance class from the centre of a site (in m)** | | | | | | | |  |
| --- | --- | --- | --- | --- | --- | --- | --- | --- | --- |
| **Distance** | 200 | 300 | 400 | 500 | 600 | 700 | 800 | 900 | 1000 |
| **Abundance** | 0.13 | **0.16** | 0.06 | 0.04 | 0.02 | 0.02 | 0.01 | -0.01 | -0.02 |
| **Richness**  **(Chao-1)** | 0.22 | **0.28** | 0.17 | 0.15 | 0.07 | 0.02 | 0.02 | 0.01 | 0.03 |

| ***Lc-diversity Index*** | **Distance class from the centre of a site (in m)** | | | | | | | |  |
| --- | --- | --- | --- | --- | --- | --- | --- | --- | --- |
| **Distance** | 200 | 300 | 400 | 500 | 600 | 700 | 800 | 900 | 1000 |
| **Abundance** | -0.18 | **-0.32** | -0.11 | -0.03 | 0.04 | 0.06 | 0.06 | 0.03 | 0.04 |
| **Richness (Chao-1)** | 0.03 | **0.20** | 0.15 | 0.19 | 0.20 | 0.20 | 0.19 | 0.20 | 0.20 |
