# Supplementary material for: Sweat bees on hot chillies: provision of pollination services by native bees in traditional slash‐and‐burn agriculture in the Yucatán Peninsula of tropical Mexico
Source: J Appl Ecol. 2017 Jan 27;54(6):1814–24. doi: 10.1111/1365-2664.12860 (PMC5697652; doi:10.1111/1365-2664.12860)
Supplement: Supplementary file 11 — Table S3. Relationships among land cover variables across sites. [file JPE-54-1814-s011.docx]

**Table S3.** **Relationships among land cover variables across sites.**

Relationships among different classes of land cover in the landscape: agricultural fallow land, home gardens and pasture (*FGP*); primary or secondary growth forest (*Forest*); cropland (*Crops*), comprising staples (maize, beans), cash crops (e.g. chilli) and orchards; and an overall index of the diversity of land cover (*Lc-diversity*). In the lower left diagonal of the box, correlation coefficients are shown (significant coefficients are in bold) and, in the upper right diagonal, the corresponding significance (probability) after correction for multiple comparisons using the False Discovery Rate (FDR) approach ([Holm 1979](#_ENREF_3); [Fox 2005](#_ENREF_2)).

|  | *FGP* | *Forest* | *Crops* | *Lc-diversity* |
| --- | --- | --- | --- | --- |
| *FGP* | - | **0.01** | 0.41 | 0.51 |
| *Forest* | **-0.69** | - | 0.34 | 0.98 |
| *Crop* | -0.21 | -0.16 | - | **0.01** |
| *Lc-diversity* Index | -0.14 | 0.01 | **0.75** | - |
