# Supplementary material for: Sweat bees on hot chillies: provision of pollination services by native bees in traditional slash‐and‐burn agriculture in the Yucatán Peninsula of tropical Mexico
Source: J Appl Ecol. 2017 Jan 27;54(6):1814–24. doi: 10.1111/1365-2664.12860 (PMC5697652; doi:10.1111/1365-2664.12860)
Supplement: Supplementary file 12 — Table S4. Statistical fit of the models of pollination success in relation to bee communities and land use across sites. [file JPE-54-1814-s012.docx]

**Table S4**. **Statistical fit of the models of pollination success in relation to bee communities and land use across sites.**

Statistical fit of models relating pollination of chilli to bee communities and surrounding land use at 11 sites; 1) combined data (abundance and species richness of bees from pan traps and transect walks), 2) abundance and species richness of bees from transect walk data only, 3) abundance and species richness of bees from pan trap data only and 4) abundance of *Lasioglossum* sp. 1 and species richness of bees from all data (from pan traps and transect walks) combined. Model fit was evaluated using SEM (structural equation models) in AMOS v. 7.0 ([Arbuckle 2006](#_ENREF_1)). Within each model we present the saturated model (with all possible hypothesized links, in which there are as many parameters estimated as degrees of freedom), and the second best fit model that corresponds to the *Forest* landscape variable in 1) and 2), *Forest* and *FGP* in 3) and *FGP* in 4), as well as, the independence (null) model, which assumes zero population covariance among the observed variables. For each of the three models, three fit indices are provided: the Akaike Information Criterion (AIC), the Root Mean Square Error of Approximation (RMSEA) and a Chi-square test (*χ^2^*).

|  | **AIC†** | **RMSEA‡** | *χ^2^* **(P value)§** |
| --- | --- | --- | --- |
| **1) Model using abundance of bees from combined data** |  |  |  |
| Independence (null) model | 257.12 | 1.14 | 0.00 |
| Best fit model *FGP* | 256.24 | 0.00 | 0.54 |
| Best fit model *Forest* | 254.02 | 0.00 | 0.58 |
| Saturated model | 20.00 | 2.14 | 0.02 |
| **2) Model using bee abundance from transect walks** | | |  |
| Independence (null) model | 257.41 | 1.23 | 0.00 |
| Best fit model *FGP* | 249.95 | 0.27 | 0.12 |
| Best fit model *Forest* | 250.00 | 0.40 | 0.04 |
| Saturated model | 42.00 | 0.00 | 0.08 |
| **3) Model using bee abundance from pan traps** | | |  |
| Independence (null) model | 252.27 | 1.30 | 0.00 |
| Best fit model *FGP* | 248.97 | 0.00 | 0.66 |
| Best fit model *Forest* | 248.97 | 0.00 | 0.60 |
| Saturated model | 42.00 | 0.00 | 0.08 |
| **4) Model using *Lasioglossum* sp. 1 abundance from combined data** | | | |
| Independence (null) model | 261.85 | 1.25 | 0.02 |
| Best fit model *FGP* | 249.90 | 0.00 | 0.72 |
| Best fit model *Forest* | 251.52 | 0.00 | 0.70 |
| Saturated model | 42.00 | 0.00 | 0.02 |

† Lower AIC values indicate better fit;

‡ RMSEA <0.08 indicates good fit;

§ *χ^2^* small *P* values indicate bad fit ([Kline 2011](#_ENREF_4))
