# Supplementary material for: Sweat bees on hot chillies: provision of pollination services by native bees in traditional slash‐and‐burn agriculture in the Yucatán Peninsula of tropical Mexico
Source: J Appl Ecol. 2017 Jan 27;54(6):1814–24. doi: 10.1111/1365-2664.12860 (PMC5697652; doi:10.1111/1365-2664.12860)
Supplement: Supplementary file 13 — Table S5. Details of bee community composition across sites. [file JPE-54-1814-s013.docx]

**Table S5.** **Details of bee community composition across sites.**

Identity, abbreviation and abundance of bee species collected at 37 chilli sites in the Yucatan Peninsula by pan trapping and transect walks (insect netting). Abbreviations correspond to the names of the species used in Figures S4, S7 and S8.

| **Family and species** | **Abbreviation** | **No. bees collected** | **% bees collected** | **No. sites at which collected** |
| --- | --- | --- | --- | --- |
| **Apidae** |  |  |  |  |
| *Ancyloscelis apiformis,* (Fabricius, 1793) | An-ap | 31 | 1.40 | 10 |
| *Apis mellifera,* Linnaeus, 1758 | Ap-me | 4 | 0.18 | 4 |
| *Centris aethyctera,* Snelling, 1974 | Ce-ae | 1 | 0.05 | 1 |
| *Centris trigonoides,* Lepeletier, 1841 | Ce-tr | 8 | 0.36 | 4 |
| *Centris* sp. 1 | Ce-sp1 | 3 | 0.14 | 3 |
| *Centris* sp. 2 | Ce-sp2 | 3 | 0.14 | 2 |
| *Centris* sp. 3 | Ce-sp3 | 16 | 0.72 | 8 |
| *Centris* sp. 4 | Ce-sp4 | 1 | 0.05 | 1 |
| *Centris* sp. 5 | Ce-sp5 | 1 | 0.05 | 1 |
| *Centris* sp. 6 | Ce-sp6 | 3 | 0.14 | 2 |
| *Cephalotrigona zexmeniae,* (Cockerell, 1912) | Cph-zx | 9 | 0.41 | 3 |
| *Ceratina capitosa,* Smith, 1879 | Ce-ca | 25 | 1.13 | 15 |
| *Ceratina* sp1 | Cer-sp1 | 189 | 8.53 | 19 |
| *Ceratina* sp. 2 | Ce-sp2 | 41 | 1.85 | 11 |
| *Ceratina* sp. 3 | Ce-sp3 | 2 | 0.09 | 2 |
| *Diadasia* sp. 1 | Di-sp1 | 1 | 0.05 | 1 |
| *Epicharis elegans,* Smith, 1861 | Epi-el | 3 | 0.14 | 2 |
| *Euglossa viridissima,* Bembé & Eltz, 2011 | Eu-vi | 7 | 0.32 | 4 |
| *Eulaema polychroma,* (Mocsáry, 1899) | El-po | 1 | 0.05 | 1 |
| *Exomalopsis* sp. 1 | Ex-sp1 | 60 | 2.71 | 14 |
| *Exomalopsis* sp. 2 | Ex-sp2 | 9 | 0.41 | 8 |
| *Exomalopsis* sp. 3 | Ex-sp3 | 13 | 0.59 | 5 |
| *Exomalopsis* sp. 4 | Ex-sp4 | 82 | 3.70 | 12 |
| *Frieseomelitta nigra* (Cresson, 1878) | Fri-ni | 62 | 2.80 | 16 |
| *Gaesischia exul*, Michener, LaBerge & Moure, 1955 | Gae-ex | 2 | 0.09 | 2 |
| *Melissodes tepaneca* Cresson, 1878 | Mel-te | 293 | 13.23 | 32 |
| *Melitoma marginella,* (Cresson, 1878) | Me-ma | 1 | 0.05 | 1 |
| *Mesoplia* sp. | Me-sp | 1 | 0.05 | 1 |
| *Nannotrigona perilampoides* (Cresson, 1878) | Na-pe | 46 | 2.08 | 8 |
| *Paratetrapedia* sp. 1 | Pa-sp1 | 5 | 0.23 | 2 |
| *Paratetrapedia* sp. 2 | Pa-sp2 | 3 | 0.14 | 2 |
| *Paratetrapedia* sp. 3 | Pa-sp3 | 1 | 0.05 | 1 |
| *Partamona bilineata (*Say 1837) | Pa-bi | 7 | 0.32 | 5 |
| *Plebeia frontalis,* (Friese, 1911) | Pl-fro | 41 | 1.85 | 13 |
| *Scaptotrigona pectoralis* (Dalla Torre, 1896) | Sca-pe | 7 | 0.32 | 4 |
| *Svastra nitida* (LaBerge, 1956) | Sr-ni | 2 | 0.09 | 1 |
| *Tetrapedia* sp. 1 | Tet-sp1 | 1 | 0.05 | 1 |
| *Triepeolus* sp. 1 | Tri-sp1 | 9 | 0.41 | 5 |
| *Trigona fulviventris,* Guérin-Méneville, 1844 | Tr-ful | 121 | 5.46 | 11 |
| *Trigona fuscipennis,* Friese, 1900 | Tr-fus | 8 | 0.36 | 1 |
| *Trigonisca pipioli,* Ayala, 1999 | Trg-pi | 6 | 0.27 | 4 |
| *Trigonisca maya,* Ayala, 1999 | Trg-my | 6 | 0.27 | 2 |
| *Xylocopa mexicanorum,* Cockerell, 1912 | Xy-me | 1 | 0.05 | 2 |
| *Xylocopa muscaria* (Fabricius, 1775) | Xy-mu | 2 | 0.09 | 1 |
| **Andrenidae** |  |  |  |  |
| *Calliopsis hondurasica,* Cockerell, 1949 | Cal-ho | 2 | 0.09 | 1 |
| *Pseudopanurgus* sp. | Ps-sp | 4 | 0.18 | 2 |
| *Pseudopanurgus* sp. 1 | Ps-sp1 | 2 | 0.09 | 1 |
| **Colletidae** |  |  |  |  |
| *Colletes* sp. 1 | Co-sp1 | 2 | 0.09 | 2 |
| *Colletes punctipennis,* Cresson, 1868 | Co-pu | 1 | 0.05 | 1 |
| *Hylaeus* sp. 1 | Hy-sp1 | 6 | 0.27 | 3 |
| *Hylaeus* sp. 2 | Hy-sp2 | 16 | 0.72 | 4 |
| *Hylaeus* sp. 3 | Hy-sp3 | 1 | 0.05 | 1 |
| **Halictidae** |  |  |  |  |
| *Augochlora aurifera,* Cockerell, 1897 | Au-au | 77 | 3.48 | 20 |
| *Augochlora cordiaefloris,* Cockerell, 1907 | Au-co | 15 | 0.68 | 8 |
| *Augochlora nigrocyanea,* Cockerell, 1897 | Au-ni | 28 | 1.26 | 15 |
| *Augochlora* sp. 1 | Au-sp1 | 9 | 0.41 | 5 |
| *Augochlora* sp. 2 | Au-sp2 | 1 | 0.05 | 1 |
| *Augochlorella* sp. 1 | Aug-sp1 | 69 | 3.12 | 10 |
| *Augochlorella* sp. 2 | Aug-sp2 | 1 | 0.05 | 1 |
| *Augochloropsis ignita* (Smith, 1861) | Aus-ig | 3 | 0.14 | 2 |
| *Augochloropsis metallica* (Fabricius, 1793) | Aus-me | 31 | 1.40 | 13 |
| *Caenaugochlora* sp. | Cau-sp | 2 | 0.09 | 2 |
| *Halictus* sp. | Ha-sp | 7 | 0.32 | 1 |
| *Halictus ligatus,* Say, 1837 | Ha-li | 2 | 0.09 | 1 |
| *Lasioglossum* sp. | La-sp | 27 | 1.22 | 1 |
| *Lasioglossum (Evylaeus)* sp. 1 | La(ev)sp1 | 12 | 0.54 | 3 |
| *Lasioglossum* sp. 1 | La-sp1 | 494 | 22.30 | 36 |
| *Lasioglossum* sp. 2 | La-sp2 | 73 | 3.30 | 21 |
| *Lasioglossum* sp. 3 | La-sp3 | 64 | 2.89 | 15 |
| *Lasioglossum* sp. 4 | La-sp4 | 17 | 0.77 | 7 |
| *Lasioglossum* sp. 5 | La-sp5 | 7 | 0.32 | 5 |
| *Lasioglossum* sp. 6 | La-sp6 | 1 | 0.05 | 1 |
| *Lasioglossum* sp. 7 | La-sp7 | 4 | 0.18 | 3 |
| **Megachilidae** |  |  |  |  |
| *Ashmeadiella* sp. 1 | As-sp1 | 1 | 0.05 | 1 |
| *Ashmeadiella sp. nova* | As-no | 2 | 0.09 | 2 |
| *Coelioxys* sp. 1 | Coe-sp1 | 2 | 0.09 | 1 |
| *Heriades* sp. | He-sp | 11 | 0.5 | 6 |
| *Heriades* sp. 1 | He-sp1 | 5 | 0.23 | 3 |
| *Megachile chichimeca,* Cresson, 1878 | Me-chi | 7 | 0.32 | 4 |
| *Megachile frugalis,* Cresson, 1872 | Me-fru | 12 | 0.54 | 1 |
| *Megachile* sp. 1 | Me-sp1 | 6 | 0.27 | 2 |
| *Megachile* sp. 2 | Me-sp2 | 14 | 0.63 | 8 |
| *Megachile* sp. 3 | Me-sp3 | 3 | 0.14 | 2 |
| *Megachile* sp. 4 | Me-sp4 | 4 | 0.18 | 4 |
| *Megachile* sp. 5 | Me-sp5 | 9 | 0.41 | 4 |
| *Megachile* sp. 6 | Me-sp6 | 2 | 0.09 | 1 |
| *Megachile* sp. 7 | Me-sp7 | 12 | 0.54 | 5 |
| *Megachile* sp. 8 | Me-sp8 | 1 | 0.05 | 1 |
| *Megachile* sp. 9 | Me-sp9 | 3 | 0.14 | 1 |
| *Megachile* sp. 10 | Me-sp10 | 3 | 0.14 | 2 |
| *Megachile* sp. 11 | Me-sp11 | 2 | 0.09 | 2 |
| **Total** |  | **2215** | **100** |  |

Species highlighted in grey were abundant (> 5%) and widespread (>10 sites) and were used in further statistical analysis.
