# Supplementary material for: Sweat bees on hot chillies: provision of pollination services by native bees in traditional slash‐and‐burn agriculture in the Yucatán Peninsula of tropical Mexico
Source: J Appl Ecol. 2017 Jan 27;54(6):1814–24. doi: 10.1111/1365-2664.12860 (PMC5697652; doi:10.1111/1365-2664.12860)
Supplement: Supplementary file 14 — Table S6. Canonical correspondence analysis of the relationships between bee communities and land use across sites. [file JPE-54-1814-s014.docx]

**Table S6**. **Canonical Correspondence Analysis of the relationships between bee communities and land use across sites.**

Results of the Canonical Correspondence Analysis (CCA) presented in Fig. S2 (supporting information), showing the percentage of variation explained by analysis of the relationships between bee diversity and three land use variables: agricultural fallow land, home gardens and pasture (*FGP*); primary or secondary growth forest (*Forest*); and crops (*Crops*). Inertia is a mean squared coefficient, which represents the total variability in species abundance with respect to the land use variables. Proportion represents the proportion of the variation explained by the data, calculated by subjecting the inertia matrix (variation of the species abundance with respect to the environment) to weighted regression. The constrained values represent the percentage of variation explained by the axes (i.e. environmental variables), in this case 14%. Here 100% represents over-fitting of the analysis and 0% represents poor explanation of the variation. However, an analysis with low constrained values still provides important information about the axes analyzed. ‘Unconstrained’ is the proportion of variation that has not been explained by the axes (i.e. environmental variables), in this case 86%. The final permutation test shows that the only variable that significantly explains the distribution of bee species is *FGP*.

|  |  | **Inertia** |  | **Proportion** | | **Rank** |  |
| --- | --- | --- | --- | --- | --- | --- | --- |
| **Total** | | 6.04 |  | 1.00 |  |  |  |
| **Constrained** |  | 0.86 |  | 0.14 |  | 4.00 |  |
| **Unconstrained** | | 5.18 |  | 0.87 |  | 32.00 |  |
| **Percentage of variation explained by the constrained axes.** | | | | | | |  |
| **Eigenvalues for constrained axes:** | | | |  |  |  |  |
| CCA1 |  | CCA2 |  | CCA3 |  | CCA4 |  |
| 0.34 |  | 0.27 |  | 0.16 |  | 0.09 |  |
| **Eigenvalues for unconstrained axes:** | | | |  |  |  |  |
| CA1 | CA2 | CA3 | CA4 | CA5 | CA6 | CA7 | CA8 |
| 0.60 | 0.51 | 0.47 | 0.41 | 0.33 | 0.32 | 0.29 | 0.27 |
| **(we show only 8 of all 32 unconstrained eigenvalues)** | | | | | |  |  |
| **Permutation test for CCA under reduced model** | | | | | |  |  |
|  | DF | AIC | F | Pr(>F) |  |  |  |
| ***FGP*** | 1 | 220.18 | 1.68 | 0.015 * |  |  |  |
| ***Lc-diversity*** | 1 | 220.22 | 1.64 | 0.075 . |  |  |  |
| ***Forest*** | 1 | 220.28 | 1.58 | 0.095 . |  |  |  |
| ***Crops*** | 1 | 220.54 | 1.32 | 0.255 |  |  |  |
| **Significance code: ‘*’ P<0.05 ‘.’ P<0.1** | | | |  |  |  |  |
