# Supplementary material for: Sweat bees on hot chillies: provision of pollination services by native bees in traditional slash‐and‐burn agriculture in the Yucatán Peninsula of tropical Mexico
Source: J Appl Ecol. 2017 Jan 27;54(6):1814–24. doi: 10.1111/1365-2664.12860 (PMC5697652; doi:10.1111/1365-2664.12860)
Supplement: Supplementary file 15 — Table S7. Relationship between bee taxa and land cover variables. [file JPE-54-1814-s015.docx]

**Table S7.** **Relationship between bee taxa and land cover variables.**

Spearman rank correlation coefficients of the relationship between bee abundance, bee species richness, the abundance of the most common species (>3%) and genera of all bees, stingless bees and non-stingless bees with the proportion of different land cover types: agricultural fallow land, home gardens and pasture (*FGP*); primary or secondary growth forest (*Forest*); crops (*Crops*); and an overall index of the diversity of land cover (*Lc-diversity*) of all three land cover classes: *FGP*, *Forest* and *Crops*, at 300 m around study sites (n = 37 sites) for all bees collected by pan trapping and transect walks. Values in bold indicate statistical significance (P<0.05). Classification of stingless and non-stingless bees and nesting habit was based on Michener (2000). After correction for multiple comparisons using the False Discovery Rate (FDR) approach ([Holm 1979](#_ENREF_3); [Fox 2005](#_ENREF_2)), only the relationship between *Forest* and bee species richness was significant.

| **Bee Taxon** |  | **Social organization** | ***FGP*** | ***Forest*** | ***Crops*** | ***Lc-diversity*** |
| --- | --- | --- | --- | --- | --- | --- |
| All bee abundance |  |  | 0.18 | 0.25 | -0.14 | -0.21 |
| All bee richness (Chao-1) |  |  | **-0.29** | ****0.50** | **-0.22** | -0.28 |
| Abundance pan traps |  |  | 0.20 | 0.02 | **-0.33** | 0.14 |
| Bee richness pan traps |  |  | -0.12 | 0.07 | **-0.31** | 0.29 |
| Abundance transects |  |  | 0.22 | -0.15 | -0.02 | -0.21 |
| Bee richness transects |  |  | **-0.22** | 0.06 | **-0.29** | 0.20 |
| **Most abundant species** | **% individuals of total individuals** |  |  |  |  |  |
| *Lasioglossum* sp. 1 | 22.30 | Solitary/Primitively eusocial | **0.40** | 0.14 | 0.01 | 0.21 |
| *Melissodes tepaneca* | 13.20 | Solitary | 0.30 | -0.17 | -0.16 | -0.16 |
| *Ceratina* sp. 1 | 8.50 | Solitary | -0.19 | **0.34** | -0.09 | -0.14 |
| *Trigona fulviventris* | 5.40 | Eusocial | 0.24 | -0.08 | **0.34** | -0.04 |
| *Exomalopsis* sp. 1 | 3.70 | Solitary,Primitively eusocial | -0.21 | 0.28 | 0.11 | -0.31 |
| *Lasioglossum* sp. 2 | 3.30 | Solitary/Primitively eusocial | 0.06 | -0.01 | -0.06 | 0.39 |
| **Most abundant genera** | **% species in genus (of all species)** |  |  |  |  |  |
| All *Megachile* abundance | 15.00 | Solitary | -0.03 | -0.07 | 0.08 | 0.05 |
| All *Lasioglossum* abundance | 10.00 | Solitary/Primitively eusocial | **0.38** | 0.11 | 0.07 | 0.04 |
| All *Centris* abundance | 9.00 | Solitary | 0.07 | 0.13 | 0.07 | 0.10 |
| All *Augochlora* abundance | 6.00 | Solitary/Primitively eusocial | -0.01 | 0.05 | 0.07 | 0.22 |
| All *Ceratina* abundance | 5.00 | Solitary | 0.23 | **0.41** | -0.05 | -0.12 |
| **Groups** | **% species in the group (of all species)** |  |  |  |  |  |
| Stingless bees | 20.88 |  | -0.05 | 0.04 | -0.20 | -0.08 |
| Non-stingless bees | 79.12 |  | 0.22 | -0.13 | -0.01 | -0.22 |
| Cavity nesters | 56.04 |  | 0.10 | 0.14 | 0.03 | -0.24 |
| Ground nesters | 43.96 |  | 0.20 | -0.22 | 0.01 | -0.10 |

Key to colours:

| r*_s_*>0.50 | Significant after correction by FDR |
| --- | --- |
| r*_s_*>0.40 | Significant, but not after correction by FDR |
| r*_s_*>0.20 | Non-significant, weak correlation |

** = *P*<0.01 after correction for FDR
