# Supplementary material for: Sweat bees on hot chillies: provision of pollination services by native bees in traditional slash‐and‐burn agriculture in the Yucatán Peninsula of tropical Mexico
Source: J Appl Ecol. 2017 Jan 27;54(6):1814–24. doi: 10.1111/1365-2664.12860 (PMC5697652; doi:10.1111/1365-2664.12860)
Supplement: Supplementary file 16 — Table S8. Statistical modelling of bee community metrics and land use across sites. [file JPE-54-1814-s016.docx]

**Table S8**. **Statistical modelling of bee community metrics and land use across sites.**

Generalised Linear Models (GLMs) for the effects of land use (*Crop, FGP, Forest*) on the bee community (abundance and richness as Chao-1 estimate) derived from either transect walk or pan trap material or combined transect walk and pan traps (total). Significant values are given in bold.

|  | **Family** | **Standardized Regression Coefficients** | **Std. Error** | ***z* value** | **Pr(>\|t\|36 DF)** |
| --- | --- | --- | --- | --- | --- |
| **Abundance (total)~** |  |  |  |  |  |
| ***Crops*** | Poisson | -0.13 | 0.03 | -1.59 | 0.11 |
| ***FGP*** | Poisson | 0.10 | 0.02 | 1.19 | 0.23 |
| ***Forest*** | Poisson | 0.43 | 0.03 | 5.54 | **<0.01 **** |
| **Abundance (transects)~** | |  |  |  |  |
| ***Crops*** | Poisson | -0.24 | 0.12 | -2.75 | **0.01 *** |
| ***FGP*** | Poisson | 0.17 | 0.05 | 2.29 | **0.05 *** |
| ***Forest*** | Poisson | 0.23 | 0.06 | 2.12 | **0.04 *** |
| **Abundance (pan traps)~** |  |  |  |  |  |
| ***Crops*** | Poisson | -0.53 | 0.10 | -9.29 | **0.01 *** |
| ***FGP*** | Poisson | 0.55 | 0.06 | 8.39 | **0.01 *** |
| ***Forest*** | Poisson | -0.42 | 0.06 | -4.88 | **0.01 *** |
| **Richness Chao-1 (total)~** |  |  |  |  |  |
| ***Crops*** | Poisson | -0.16 | 0.05 | -4.23 | **<0.01 **** |
| ***FGP*** | Poisson | -0.28 | 0.05 | -7.12 | **<0.01 **** |
| ***Forest*** | Poisson | 0.25 | 0.04 | 7.01 | **<0.01 **** |
| **Richness (Chao-1) transects~** |  |  |  |  |  |
| ***Crops*** | Poisson | -0.13 | 0.07 | -1.82 | 0.08 . |
| ***FGP*** | Poisson | 0.07 | 0.03 | 0.35 | 0.73 |
| ***Forest*** | Poisson | <0.01 | 0.03 | 0.02 | 0.98 |
| **Richness (Chao-1) pan traps~** |  |  |  |  |  |
| ***Crops*** | Poisson | -0.36 | 0.07 | -1.52 | 0.13 |
| ***FGP*** | Poisson | -0.10 | 0.03 | -0.33 | 0.74 |
| ***Forest*** | Poisson | 0.26 | 0.03 | 0.86 | 0.39 |
