# Supplementary material for: Sweat bees on hot chillies: provision of pollination services by native bees in traditional slash‐and‐burn agriculture in the Yucatán Peninsula of tropical Mexico
Source: J Appl Ecol. 2017 Jan 27;54(6):1814–24. doi: 10.1111/1365-2664.12860 (PMC5697652; doi:10.1111/1365-2664.12860)
Supplement: Supplementary file 17 — Table S9. Experimental estimates of pollination service provision across sites. [file JPE-54-1814-s017.docx]

**Table S9. Experimental estimates of pollination service provision across sites.**

Experimentally derived Pollinator Service Provision (*PSP*) index for chilli at 11 sites based on the number of seeds per fruit (seed) and fruit weight (weight). *PSP* values above 1 indicate extremely successful open pollination coupled with less efficient cross pollination at a site.

| **Site number** | **Site Name** | ***PSP* (seed)** | ***PSP* (weight)** |
| --- | --- | --- | --- |
| 4 | Homún | 0.95 | 0.87 |
| 18 | Tixcacaltuyub | 0.48 | 0.54 |
| 19 | Timul A | 0.75 | 0.84 |
| 20 | Nenela C | 0.75 | 0.69 |
| 24 | Nenela A | 1.23 | 1.06 |
| 25 | Tah Dziú B | 0.57 | 0.62 |
| 26 | Tah Dziú A | 0.52 | 0.63 |
| 29 | Tixcuytun A | 0.92 | 1.03 |
| 31 | Tixcuytun B | 0.45 | 0.45 |
| 32 | Tixcuytun C | 0.76 | 0.81 |
| 36 | Yaxcopil | 0.56 | 0.69 |
