# Supplementary material for: Sweat bees on hot chillies: provision of pollination services by native bees in traditional slash‐and‐burn agriculture in the Yucatán Peninsula of tropical Mexico
Source: J Appl Ecol. 2017 Jan 27;54(6):1814–24. doi: 10.1111/1365-2664.12860 (PMC5697652; doi:10.1111/1365-2664.12860)
Supplement: Supplementary file 18 — Table S10. Statistical modelling of bee communities with pollination service provision. [file JPE-54-1814-s018.docx]

**Table S10**. **Statistical modelling of bee communities with pollination service provision.**

Linear models (LMs) for the effects of bee abundance and richness, from the combined dataset (pan traps and transect walks, as Total), data from transects alone (Transects), and data from pan traps alone (Pan traps), on the index of experimental Pollination Service Provision (*PSP*).

|  | **Family** | **R-squared** | **Standardized Regression Coefficients** | **Std. Error** | ***z/t* value** | **Pr(>\|t\|8 DF)** |
| --- | --- | --- | --- | --- | --- | --- |
| ***PSP*~ (Total)** |  | **0.49** |  |  |  |  |
| **Abundance** | Gaussian |  | 0.78 | 0.05 | 3.42 | **<0.01 **** |
| **Richness (Chao-1)** | Gaussian |  | -0.15 | 0.05 | -0.67 | 0.52 |
| ***PSP*~ (Transects)** |  | **0.12** |  |  |  |  |
| **Abundance transect** | Gaussian |  | 0.56 | 0.07 | 1.82 | 0.11 |
| **Richness transect** | Gaussian |  | -0.17 | 0.07 | 0.54 | 0.60 |
| ***PSP*~ (Pan traps)** |  | **0.65** |  |  |  |  |
| **Abundance pan traps** | Gaussian |  | 0.97 | 0.05 | 4.42 | **<0.01 **** |
| **Richness pan traps** | Gaussian |  | -0.65 | 0.05 | -2.96 | **0.02 *** |
