# Supplementary material for: Sweat bees on hot chillies: provision of pollination services by native bees in traditional slash‐and‐burn agriculture in the Yucatán Peninsula of tropical Mexico
Source: J Appl Ecol. 2017 Jan 27;54(6):1814–24. doi: 10.1111/1365-2664.12860 (PMC5697652; doi:10.1111/1365-2664.12860)
Supplement: Supplementary file 19 — Table S11. Statistical modelling of the most abundant bees with pollination service provision. [file JPE-54-1814-s019.docx]

**Table S11**. **Statistical modelling of the most abundant bees with pollination service provision.**

Linear model (LMs) for the effects of the most abundant species, according to collection method, on the index of experimental Pollination Service Provision (*PSP*): 1) combined (pan trap and transect walk) data: *Lasioglossum* sp. 1, *Melissodes tepaneca,* *Ceratina* sp. 1 and *Trigona fulviventris* abundance; 2) data from transect walks alone: *Ceratina* sp1, *Frieseomelitta nigra* and *Lasioglossum* sp1 abundance; and 3) data from pan traps alone: *Ceratina capitosa* and *Lasioglossum* sp1 abundance. All LMs were simplified by backward stepwise selection based on the Akaike Information Criterion (AIC). All predictors were check for collinearity.

|  | **Family** | **R-squared** | **Standardized Regression Coefficients** | **Std. Error** | ***z/t* value** | **Pr(>\|t\|7 DF)** |
| --- | --- | --- | --- | --- | --- | --- |
| ***PSP*~ (Total)** |  | **0.57** |  |  |  |  |
| *Ceratina* sp. 1 | Gaussian |  | -0.28 | 0.05 | -1.26 | 0.25 |
| *Lasioglossum* sp. 1 | Gaussian |  | 0.74 | 0.05 | 3.34 | **0.01 *** |
| *Melissodes tepaneca* | Gaussian |  | -0.09 | 0.05 | -0.40 | 0.71 |
| *Trigona fulviventris* | Gaussian |  | -0.15 | 0.06 | -0.64 | 0.55 |
| ***PSP*~ (Transects)** |  | **0.58** |  |  |  |  |
| *Ceratina* sp. 1 | Gaussian |  | -0.13 | 0.06 | -0.53 | 0.61 |
| *Frieseomelitta nigra* | Gaussian |  | 0.42 | 0.06 | 1.70 | 0.13 |
| *Lasioglossum* sp. 1 | Gaussian |  | 0.75 | 0.05 | 3.20 | **0.02** * |
| ***PSP*~ (Pan traps)** |  | **0.69** |  |  |  |  |
| *Ceratina capitosa* | Gaussian |  | -0.17 | 0.05 | -0.81 | 0.44 |
| *Lasioglossum* sp. 1 | Gaussian |  | 0.86 | 0.01 | 4.21 | **<0. 01 **** |
