# Supplementary material for: Sweat bees on hot chillies: provision of pollination services by native bees in traditional slash‐and‐burn agriculture in the Yucatán Peninsula of tropical Mexico
Source: J Appl Ecol. 2017 Jan 27;54(6):1814–24. doi: 10.1111/1365-2664.12860 (PMC5697652; doi:10.1111/1365-2664.12860)
Supplement: Supplementary file 20 — Table S12. Statistical modelling of land use with pollination service provision. [file JPE-54-1814-s020.docx]

**Table S12**. **Statistical modelling of land use with pollination service provision.**

Linear model (LM) for the effects of land use (*Crops, FGP, Forest*) on Pollination Service Provision (*PSP*).

|  | **Family** | **Standardized Regression Coefficients** | **Std. Error** | ***z/t* value** | **Pr(>\|t\|8 DF)** |
| --- | --- | --- | --- | --- | --- |
| **PSP~** |  |  |  |  |  |
| ***Crops*** | Gaussian | <0.01 | <0.01 | 0.08 | 0.99 |
| ***FGP*** | Gaussian | 0.44 | 0.07 | 1.46 | 0.18 |
| ***Forest*** | Gaussian | -0.44 | 0.07 | -1.48 | 0.17 |
